# Experiences of interventions aiming to improve the mental health and well‐being of children and young people with a long‐term physical condition: A systematic review and meta‐ethnography

**DOI:** 10.1111/cch.12708

**Published:** 2019-08-16

**Authors:** Liz Shaw, Darren Moore, Michael Nunns, Jo Thompson Coon, Tamsin Ford, Vashti Berry, Erin Walker, Isobel Heyman, Christopher Dickens, Sophie Bennett, Roz Shafran, Ruth Garside

**Affiliations:** ^1^ University of Exeter Medical School Exeter UK; ^2^ Graduate School of Education University of Exeter Exeter UK; ^3^ Evidence Synthesis & Modelling for Health Improvement University of Exeter Medical School Exeter UK; ^4^ Child Mental Health Research Group University of Exeter Medical School Exeter UK; ^5^ Centre for Outcomes and Experience Research in Children's Health, Illness and Disability, Great Ormond Street Hospital London UK; ^6^ Great Ormond Street Institute of Child Health University College London London UK; ^7^ Psychological Medicine University of Exeter Medical School Exeter UK; ^8^ European Centre for Environment and Human Health University of Exeter Medical School Truro UK

**Keywords:** children, intervention, long‐term condition, mental health, qualitative research methods, systematic review, well‐being

## Abstract

**Background:**

Children and young people with long‐term physical health conditions are at increased risk of experiencing mental health and well‐being difficulties. However, there is a lack of research that explores the experiences of and attitudes towards interventions aiming to improve their mental health and well‐being. This systematic review seeks to address this gap in the literature by exploring what children and young people with long‐term conditions, their caregivers, and health practitioners perceive to be important aspects of interventions aiming to improve their mental health and well‐being.

**Methods:**

An information specialist searched five academic databases using predefined criteria for qualitative evaluations of interventions aiming to improve the mental health or well‐being of children with long‐term physical conditions. Reviewers also performed supplementary citation and grey literature searches. Two reviewers independently screened titles, abstracts, and full texts that met the inclusion criteria and conducted data extraction and quality assessment. Meta‐ethnography was used to synthesize the findings.

**Results:**

Screening identified 60 relevant articles. We identified five overarching constructs through the synthesis: (a) Getting In and Staying In, (b) Therapeutic Foundation, (c) Social Support, (d) A Hopeful Alternative, and (e) Empowerment. The line of argument that links these constructs together indicates that when interventions can provide an environment that allows young people to share their experiences and build empathetic relationships, it can enable participants to access social support and increase feelings of hope and empowerment.

**Conclusion:**

These findings may provide a framework to inform the development of mental health interventions for this population and evaluate existing interventions that already include some of the components or processes identified by this research. Further research is needed to establish which of the constructs identified by the line of argument are most effective in improving the mental well‐being of young people living with long‐term conditions.

**Key Messages**

- The line of argument presented within this paper explores the relationship between the five constructs developed through the qualitative evidence synthesis: “Getting In and Staying In,” “A Therapeutic Foundation,” “Social Support,” “Hope and Inspiration,” and “Empowerment.”
- Social support from peers and the people delivering the intervention appear to play a key role in supporting CYP to access an intervention, gain new skills, and develop an alternative, more hopeful view for the future.
- Ensuring that interventions are sustainable, accessible, and relevant to CYP's mental health needs can create the opportunity for CYP to engage with the therapeutic potential of an intervention.
- A Therapeutic Foundation where CYP feel safe, valued, and able to express themselves is a core component of mental health interventions.
- The interaction between feelings of increased hope, empowerment, and improved social support may help sustain improvements to CYP's mental health beyond the end of the intervention.
- Process evaluations should be incorporated alongside randomized controlled trials to explore which interventions are effective and for whom.

## 1 INTRODUCTION

Children and young people (CYP) living with long‐term physical health conditions (LTCs) are up to four times more likely to experience mental health or well‐being difficulties than their physically healthy peers (Hysing, Elgen, Gillberg, Lie, & Lundervold, 2007; Pinquart & Shen, 2011). The negative impact on quality of life of mental health problems alone can be as great as, or even more significant than, the impact of the LTC (Baca, Vickrey, Caplan, Vassar, & Berg, 2011). Mental ill health can also exacerbate physical health symptoms, which may then increase the risk of poorer long‐term outcomes, such as symptom control and medication adherence (Lustman & Clouse, 2005).

Between £8 and £13 billion of National Health Service (NHS) spending in England is linked to co‐morbid mental health problems among adults and children with LTCs (Naylor et al., 2012). Psychological interventions can reduce healthcare costs associated with inpatient hospital admissions by up to 20% (Chiles, Lambert, & Hatch, 1999; Naylor et al., 2012). This highlights the importance of access to timely, evidence‐based mental health and well‐being treatments for people living with LTCs. Unfortunately, there is a paucity of research examining the effectiveness of psychological interventions for alleviating symptoms of mental ill health, such as anxiety or depression, amongst CYP with LTCs (Bennett, Shafran, Coughtrey, Walker, & Heyman, 2015). Existing trials are underpowered and of low quality, with heterogeneity in intervention design and outcome measures limiting the confidence, which can be placed in research findings (Bennett et al., 2015; Moore et al. 2019).

In addition to research evaluating the effectiveness of interventions aiming to improve the mental health of CYP with LTCs, understanding the experiences and perceptions of CYP and their families and the people delivering interventions is a crucial step in understanding the impact of existing interventions on the child themselves, as well as the family and social system around them. Patient and practitioner views provide a valuable addition to quantitative measures of effectiveness by identifying factors that may act as barriers or facilitators to implementing evidence based medicine (Green & Britton, 1998). Patient attitudes and experiences also offer valuable information regarding the wider context in which health services are delivered (Lorenc, Pearson, Jamal, Cooper, & Garside, 2012) and should inform the development and delivery of new health services (Department of Health, 2015; NHS England, 2014).

Although qualitative evidence syntheses have been conducted that explore CYP's experience of their LTCs and the impact on their lives (Lum et al., 2017; Venning, Eliott, Wilson, & Kettler, 2008), none have examined the views of key stakeholders on interventions aiming to improve the well‐being of CYP with LTCs, despite the existence of primary qualitative research within this area. Here, we aimed to explore the views and experiences of CYP with LTCs, their caregivers, and practitioners of interventions intended to improve the mental health and well‐being of CYP with LTCs. We utilize a systematic review methodology to address this aim in answer to a specific call from the National Institute of Health Research. This article reports the relationship between the key constructs arising from the evidence identified by the qualitative systematic review conducted by Moore et al (2019) and aims to provide an accessible summary of the factors CYP, their parents, and professionals identify as being important within mental health and well‐being interventions for CYP with LTCs.

### 1.1 Key definitions

CYP were defined as individuals aged from birth to 25 years. We considered several of the most commonly used definitions in the literature (Perrin et al., 1993; Pless & Douglas, 1971;Stein, Bauman, Westbrook, Coupey, & Ireys, 1993) to define long‐term physical health conditions as “diagnosed physical health conditions, with an expected duration of at least three months, where cure is considered unlikely, causing limitations in ordinary activities and necessitating medical care, or related services, beyond what is usual for age in question.” 1This was the working definition used for this review. We recognize that for some LTCs, such as cancer, 5‐year survival rates are increasing. CYP with learning/intellectual disabilities were excluded from this review as this population group require additional adaptation to both intervention content and delivery, making the review's scope too broad to incorporate findings into a meaningful synthesis. This review considers the term *mental health* to encompass more than the absence of psychiatric disorders to include the concepts of adjustment, well‐being, and coping, as is consistent with other well‐known definitions of mental health (Keyes, 2005;Nastasi & Borja, 2016). The current review was, therefore, not restricted to samples with emotional or behavioural symptoms.

## 2 METHODS

The review protocol was registered on PROSPERO (doi:http://10.0.59.20/CRD42015027353), conducted according to best practice guidelines (Centre for Reviews and Dissemination, 2009) and reported according to the ENTREQ statement (Tong, Flemming, McInnes, Oliver, & Craig, 2012).

### 2.1 Search strategy and article selection

The pre‐planned strategy used controlled headings (e.g., MeSH) and free text searching. Search terms were grouped according to four concepts: CYP, mental health, long‐term physical conditions, and type of qualitative study design or analysis (See Appendix A for an example search strategy used within PsycINFO). An information specialist (M. R.) entered the search terms into five electronic databases: MEDLINE (including MEDLINE in‐process), PsycINFO, CINAHL, HMIC, and Conference Proceedings Citation Index, on April 19, 2016, and searched the website OpenGrey via http://www.opengrey.eu/) on June 23, 2016. Two reviewers (L. S. and M. N.) and an information specialist (J. T. B.) supplemented the electronic searches with forward and backward citation chasing based upon included studies and other key reviews identified through the search process. A CYP advisory group and clinical topic experts (see Moore et al., 2019) identified 46 websites (see Appendix B), which were searched for primary qualitative research.

A group of eight reviewers (L. S., D. M., M. N., I. R., J. T. B., J. T. C., M. R., and V. B.) shared independent screening of the titles and abstracts of the identified records, with two reviewers screening each article against the predetermined inclusion criteria outlined in Table 1. Full texts of potentially eligible articles were screened in the same way. Disagreements were resolved through discussion between two members of the core review team (D. M., M. N., and L. S.). Reviewers recorded screening decisions within Endnote X7.

**Table 1:** Inclusion and exclusion criteria

Criteria	Specification
Population	Included if
	○ CYP aged ≤25 years old with any LTC,
	○ the parents and families of CYP aged ≤25 years old with any LTC,
	○ those involved in the delivery of interventions to improve mental health and well‐being in CYP with LTCs.
	Excluded if
	○ LTC was obesity,
	○ all participants had learning/intellectual disabilities (i.e., IQ < 70).
Intervention	Included if
	○ intervention aimed to improve CYP's mental health and well‐being,
	○ intervention targeted CYP's mental health directly (i.e., CYP were recipients) or indirectly (e.g., parenting interventions),
	○ attitudes towards an intervention in development or interventions they chose not to receive.
	Exclude if
	○ focus on mental health *service provision* rather than specific interventions.
Outcomes	Include
	○ attitudes, experiences, perceptions, and understanding of CYP with LTCs, their parents, or the practitioners who have delivered such interventions regarding interventions focused on the mental health/well‐being of CYP with LTCs.
Study design	Include if
	○ qualitative data collection, e.g., interviews and focus groups,
	○ qualitative data analysis, e.g., thematic analysis, framework analysis, and constant comparative method,
	○ may be stand‐alone qualitative research or reported as part of a mixed methods intervention evaluation or process evaluation.
	Exclude if
	○ qualitative data only provided through open‐ended questionnaire items.
Country and language	Include if
	○ full text is in English,
	○ Organisation for Economic Coorperation and Development (OECD) setting.

Abbreviations: CYP, children and young people; LTC, long‐term physical health condition.

### 2.2 Data extraction and critical appraisal

Four reviewers' (L. S., M. N., I. R., and D. M.) extracted data summarizing each article's aims, participants, methods, intervention, findings, and study quality into Microsoft Office Excel 2010. D. M. or L. S. checked each extraction, with disagreements resolved through discussion. The 13 quality appraisal items used were based upon the Wallace checklist (Wallace, Croucher, Quilgars, & Baldwin, 2004). We added an additional question: “Are the interventions of interest clearly described?” based upon a previous review on intervention experience (Moore et al., 2016). Article quality was not used as a basis for inclusion in the review but informed the confidence reviewers could place in the synthesis findings. Greater weight was given to papers containing a higher quantity of more conceptual or interpretative data within the synthesis, consistent with other systematic reviews using meta‐ethnography (Cochrane Methods: Qualitative and Implementation, 2018).

### 2.3 Data synthesis

Reviewers synthesized the data using meta‐ethnography (Noblit & Hare, 1988). This approach has been applied in health research for interpreting findings across qualitative studies (e.g., Frost, Garside, Cooper, & Britten, 2014) and permits the comparison of different data sets, with the interpretation incorporated throughout the analytical process resulting in a final synthesis that constitutes more than “a sum of its parts” (Barnett‐Page & Thomas, 2009, p. 8). Meta‐ethnography consists of four stages of analysis:

1. Reading and rereading the included studies to ensure that reviewers were familiar with each article

This phase began during study selection and continued throughout data synthesis.

- Determining how the studies were related

Structured summaries enabled comparisons of information across different articles (Frost et al., 2014). Summaries included the following information: participant's perspective, type of LTC, mental health intervention target (e.g., procedural anxiety, depression, and coping), and any additional non‐mental health intervention targets (e.g., LTC management and social skills).

- Reciprocal translation of studies

L. S. extracted quotes and author interpretation of the material provided by study participants, known as first‐ and second‐order construct data (Atkins et al., 2008), from the results and discussion of each article. L. S. compared this data across included studies and developed concept maps to show the relationships between analytical ideas and themes. These were checked by a second reviewer (D. M). L. S. used NVivo v.11 software to conduct line‐by‐line coding, against a framework developed from these concept maps. This process identified new ideas that were either not included or conflicted with the initial coding framework, meaning each included article was re‐examined in an iterative cycle of analysis.

Reviewers began by synthesizing articles containing two or more pages of conceptually rich data. This prioritized articles that had the potential to have the greatest influence on developing constructs. The reviewers then used a maximum variation purposive sampling strategy (Suri, 2011) to introduce articles containing less interpretive data into the reciprocal translation process. For detail regarding the purposive sampling approach, see Table S1 and Appendix C.

- Synthesizing translations/creating a line of argument

Reviewers initially synthesized the views of CYP, parents, and practitioners separately to ensure any differences in the views and/or experiences between these groups were identified. L. S. and D. M. also considered the experiences of interventions targeting procedural anxiety separately (see Appendix C). Due to the similarity between themes emerging from these populations, themes for each individual group were not reported. Reviewers considered the influence of CYP's age, LTC, and type of intervention received during theme development.

L. S. grouped related themes together to form five overarching constructs, which were checked by two reviewers (D. M. and R. G.) and reported in full within the project report (Moore et al., 2019). Each overarching construct was informed by at least 10 articles that achieved 10 or more positive ratings on the Wallace checklist (Wallace et al., 2004). A line of argument model was developed that describes the relationships between each overarching construct and the themes contributing to them.

## 3 RESULTS

**Figure 1:**
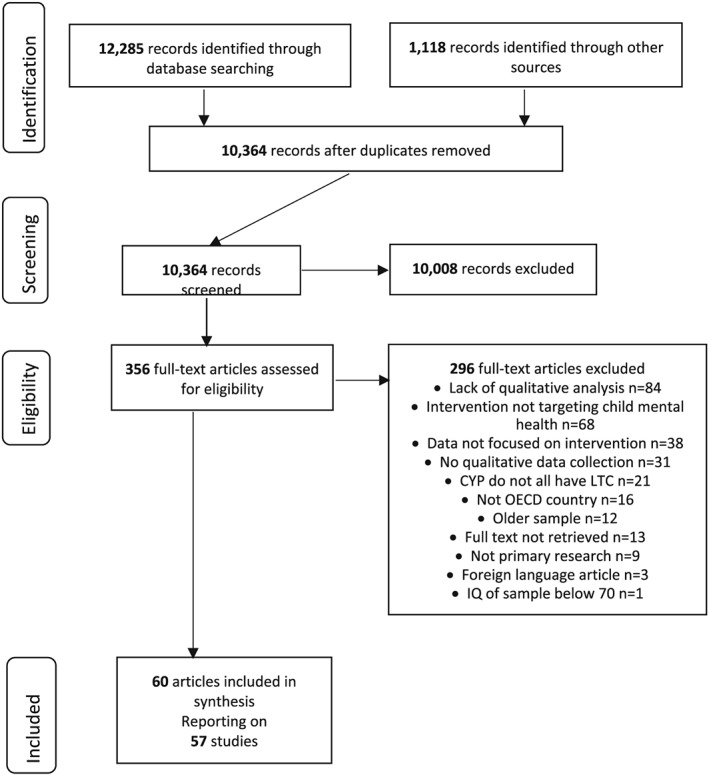
PRISMA diagram

Database searches identified 12,285 records, with 1,118 additional records found through a combination of grey literature searches, citation chasing, and reference list searches of relevant reviews. Following the screening process outlined in the Preferred Reporting Items for Systematic Reviews and Meta Analysis (PRISMA) guidelines (see Figure 1), 60 articles (from 57 studies) were eligible for inclusion (see Appendix D). Articles from the same study typically described intervention experiences in different levels of detail, influencing the stage they entered in the reciprocal translation process (Table S1).

### 3.1 Study characteristics

The 57 included studies were published between 1991 and 2016 and conducted in seven different countries, the most common being the USA (*n* = 25), Canada (*n* = 13), and the United Kingdom (*n* = 9).

The most common data collection strategies utilized within the primary studies were interviews (*n* = 48), focus groups (*n* = 5), or analysis of posts made within online discussion forums (*n* = 4). Median sample size was 23 participants (range 3–100). Twenty studies included only CYP, one study collected data from only the practitioners delivering the intervention, and four studies collected data from parents alone. The remaining studies included views from participants from more than one participant group. The most common types of intervention were categorized by reviewers (D. M., L. S., and M. N.) as Online support interventions (*n* = 12), Camps (*n* = 9), and Music therapy (*n* = 6). Table S1 provides detail regarding the focus of each study, method of analysis, and intervention type for each study.

The most common type of LTC were cancer (*n* = 14), HIV (*n* = 8), and a mix of LTCs (*n* = 10). Interventions focused on improving aspects of emotional well‐being, such as coping (*n* = 26), self‐esteem (*n* = 13), or providing emotional support (*n* = 11), although symptoms of mental ill health were not routinely measured. Other intervention aims included improving physical symptoms relating to the LTC and social skills. Table S2 outlines the structure and setting of each intervention.

Only 13 articles scored positively on at least 12 quality appraisal questions (see Table S3). Articles typically described their data collection method, had clear research questions, used an appropriate study design, and had findings supported by data. However, the theoretical or ideological perspective of the author was not reported in 45 articles; thus, its influence upon the study design, methods, or research findings could not be determined.

### 3.2 Line of argument

Five overarching constructs arose from the translation of first‐ and second‐order construct data across all 60 articles: “Getting In and Staying In,” “A Therapeutic Foundation,” “Social Support,” “A Hopeful Alternative,” and “Empowerment.” Table 2 provides examples of participant quotes supporting each construct, with Table S4 providing details of the articles which support each subtheme.

**Table 2:** Constructs contributing to line of argument

Overarching construct	Second‐order construct data (author interpretation extracted from articles included in review)	Supporting quotes
Getting In and Staying In	Clearly, greater availability of a range of educational materials and group or individual interventions combined with ease of access will be of paramount importance in the future (Barlow, Shaw, & Harrison, 1999).	“They said they only had one social worker for the outpatient department and it was all very difficult for them to see him” [Parent, ] (Lewis, Klineberg, Towns, Moore, & Steinbeck, 2016, p. 2546).
	The habitat of fun consisted of abundance and opportunities for transgressions, which were grounded in an unceasing focus on campers' enjoyment and engagement (, Witt, & Watts, 2011). Again, some participants would have changed the meeting time to make it more convenient for families (Brodeur, 2005).	“I think [this camp is] unique because you are in a setting with so many kids, and you are in a setting where you are scheduled to have fun constantly and you do not have as much down time as at home, so it creates a habitat of fun and constantly going that you do not get anywhere else.” [Camp counsellor, LTC] (Gillard et al., 2011). “The only thing I would change, and that's from having older kids and mine were older than most of the others, would have been a weekend or a Friday night because of the homework and getting downtown and coming back. That was pretty much a 2–1/2 hour, 3‐hour venture by the time we left and came back. Now that I'm working, it would be almost impossible” [Parent,] (Brodeur, 2005, p. 242).
Therapeutic Foundation	Children and parents said how important it was that parents support their child during needle‐procedures. They provided children with familiarity, reassurance, security, and practical support. (Ayers, Muller, Mahoney, & Seddon, 2011)	“If I actually knew the nurse it would be okay, but if I did not I would be like ‘what's going on? I do not even know you!' ” [CYP, Cystic Fibrosis] (Ayers et al., 2011 p. 337). “And I personally would not have coped very well if they had just invited me to go to somewhere. I needed my mum there” [CYP, CFS]. (Dennison, Stanbrook, Moss‐Morris, Yardley, & Chalder, 2010, p. 175).
	Feeling like there are others who share experiences, who talk about topics that cannot be discussed elsewhere and where one feels that they are just like everyone, are important for all children and teens. Feeling “normal” is even more important when one lives with a condition that is often kept secret due to associated shame and stigma (Muskat, Salter, Shindler, Porter, & Bitnun, 2016).	“My family, not really, and outside like friends no, cause you do not really know who to trust. And even in my family it's awkward to talk about but here I know I can talk about it with these people and that's really good. I like feeling that I can talk to someone, it's really good, yeah … Here you feel like it's not taboo, you know” [CYP, HIV] (Muskat et al., 2016, p. 8).
	Campers expressed feeling a sense of love, respect, happiness, and caring throughout their time at camp ( et al. 2006).	“I would take from camp the vibe that I get – the vibe of caring and respect and love that is just emanating through everything, through every activity in the cabins and everywhere” [CYP, Mixed LTCs] (Gillard et al., 2016, p. 116).
Social Support	Several participants talked about how they felt the program had brought their family closer together (Brodeur, 2005). Loneliness and social dissatisfaction decreased (Stewart, Letourneau, Masuda, Anderson, & McGhan, 2013a).	“We realized the necessity just to set aside time. How well we do it is a different story. But realized the necessity to sort of just set everything aside and just be together and play Monopoly or whatever” [Parent, ] (Brodeur, 2005, p. 170). “It was nice for her to see that other people were in her shoes, that what I think was the best part, to see that other people are dealing with the same things she was dealing with. You do not feel like you are alone” [Parent, Allergies/Asthma] (Stewart et al., 2013a, p. 181).
	The mentors told them about their troubles and fears (at group meetings too) and the mentees were amazed to see diabetics, who had the same fears as their own, managing to function well and share their feelings, difficulties, and successes. This experience provided a sense of relief, optimism and hope (Bartnetz et al., 2012).	“You can share deep experiences and fears that other people will not understand or do not know how to calm” [CYP, T1D] (Barnetz et al.,^2012^, p. 475). “Helped us to discuss concerns and feelings that otherwise might not surface” [Parent, ABI] (Gan, Gargaro, Kreutzer, Boschen, & Wright, 2010, p. 659).
A Hopeful Alternative	The mentees admired their mentors. Numerous mentees reported adopting and emulating parts of their mentors' behaviour regarding diabetes (Barnetz, 2012).	“It was fun like it's never been before, I saw someone who is in control of the situation and is not afraid of diabetes. If he can do it, I've got to succeed....I'm less afraid because of the project; I met people there with amazing abilities” [CYP, T1D] (Barnetz, 2012, p. 474).
	When asked what young HIV‐positive women need to make healthier life choices and decrease risky behaviors, participants emphasized the need for comprehensive programs that extended beyond HIV specific topics. They requested programs that address a wide range of issues impacting their lives such as self‐esteem, self‐confidence, self‐worth, living with HIV, sexuality, coping mechanisms, handling adversity, and developing and maintaining healthy relationships (Hosek et al., 2012).	“My perfect program would not just not only be focused on the infection. It would just really be building self‐worth, building self‐esteem like all the way around. So many youth have a hard time just making that transition perhaps to the college, and still be like do they have to take care of themselves, either by nutrition …” [CYP, HIV] (Hosek et al., 2012, p. 293).
	During the program, all participants noted that they had increased their overall daily physical activity, incorporated planned physical activities into their daily schedules (Table 7), and experienced increased motivation to go out or be with their friends even if they had pain (Kashikar‐Zuck et al., 2016).	“I used activity pacing did not push myself too far and I was still able to stay with my friends and do what they were doing” [CYP, LTC] (Kashikar‐Zuck et al., 2016, p. 74).
	They also saw themselves as able to give information to help other children (Bluebond‐Langer, Perkel, & Goertzel, 1991).	“Well, Kim is on the kind of therapy now that just finished, so now she can come to me and ask me what it's like, and I can tell her” [CYP, Cancer] (Bluebond‐Langer et al., 1991, p. 75).
Empowerment	Several participants also discussed the benefits of skills‐based education and learning strategies to manage diabetes in public (Serlachius, Northam, Frydenberg, & Cameron, 2012).	“Like strategies, or ways to deal with the public thing. Like developing a skill where you stop worrying about what strangers would think, for example at the footy” [CYP, T1D] (Serlachius et al., 2012, p. 319).
	As part of the rigorous program within the camp, participants realized success in mastering difficult tasks (Tiemens, Beveridge, & Nicholas, 2007).	“I was like, ‘wow' you know, hard things that you overcome, you are, kind of feel that you are such a good person” [CYP, CFD] (Tiemens et al., 2007, p. 65).
	Initial uncertainty or reservation was replaced by a sense of pride, competence, and mastery (Burns, Robb, Phillips‐Salimi, & Haase, 2010).	“So it was that short little brief of ‘I am in control … this is my project' was really, really good for him” [Parent, Cancer] (Burns et al., 2010, e24).

Abbreviations: ABI, acquired brain injury; CF, cystic fibrosis; CFD, craniofacial differences; CFS, chronic fatigue syndrome; CHD, congenital heart disease; CP, cerebral palsy; T1D, Type 1 diabetes.

**Figure 2:**
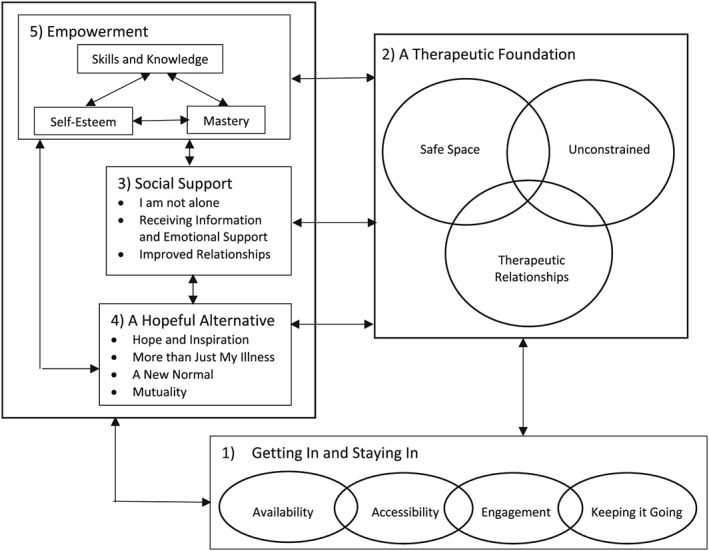
Line of argument

Figure 2 conceptualizes the line of argument. A “Therapeutic Foundation” is essential to enable CYP and their families to achieve benefit from an intervention. “A Therapeutic Foundation” may support CYP and their families to access and provide Social Support as conceptualized within construct 3, which can help CYP living with physical health conditions to feel less isolated and contribute towards feelings of safety, being valued, and unconstrained as described within construct 2.

Being able to access A Therapeutic Foundation and/or Social Support can help CYP develop positive self‐esteem and learn new or practise existing skills, which over time can contribute towards feelings of empowerment (construct 5). The interaction between experiencing A Therapeutic Foundation, receiving Social Support and experiencing Empowerment can also help create a sense of hope for CYP, generated through meeting peers who are living fulling lives alongside their LTC, taking part in everyday activities, and contributing positively within their social relationships, as illustrated by ‘A Hopeful Alternative‘.

Although some of the concepts discussed within each construct may be more pertinent at certain stages of an intervention, the relationships between constructs 2, 3, and 4 are bidirectional and self‐reinforcing and promote continued engagement with the intervention. The longer CYP and families engage with an intervention, the more they are likely to develop therapeutic relationships with intervention deliverers and/or other participants and experience the therapeutic benefits associated with the intervention. Thus, the therapeutic effect of these bidirectional relationships could be regarded as accumulative over time. ‘Getting In and Staying In' describes factors that may influence the availability and accessibility of an intervention as well as the ongoing engagement of people taking part and discusses how the social relationships, increased sense of empowerment, and hope developed through taking part in an intervention are key to prolonging its benefits beyond its endpoint.

### 3.3 Construct

#### 3.3.1 Getting In and Staying In

This construct conceptualizes CYPs ongoing interaction with an intervention aimed at improving their mental health: from the initial recognition of their mental health need(s) and support from professionals, maintaining engagement over time, and retaining the effects of the intervention after it has ended.

To make interventions available to those who need them, service providers should ensure they have adequate funding for equipment (e.g., Nicholas et al., 2007), staff expertise in intervention design and delivery (Whittemore, Grey, Lindemann, Ambrosino, & Jaser, 2010), and space to stage an intervention ( et al., 2016). Health and social care staff play a key role in recognizing the mental health needs of CYP and supporting them and their families to access an intervention that is appropriate for their needs (e.g., Desai, Sutton, Staley, & Hannon, 2014;Fair, Connor, Albright, Wise, & Jones, 2012;Lewis et al., 2016). As discussed within A Therapeutic Foundation below, CYP find it validating when their emotions and experiences are recognized by others. This suggests that contact with health and social care practitioners who can recognize the impact of an LTC on CYPs mental health and offer some emotional support may have a beneficial effect on CYPs mental well‐being before they even access a more formalized mental health intervention.

Recognizing the social and family context of CYP was key to ensure they had the appropriate social support available to enable them to maintain engagement with an intervention over time. Parents highlighted the importance of flexibility in the delivery of interventions, such as considering the time of day (e.g., Brodeur, 2005) and setting (e.g., Muskat et al., 2016). Providing opportunities for CYP to maintain the peer relationships formed during an intervention via social media (e.g., Kirk & Milnes, 2016) or organized events (e.g., Desai et al., 2014; Lewis et al., 2016) may help maintain the beneficial effects of the social support received through an intervention (as discussed within the construct Social Support) beyond its scheduled endpoint.

Consideration of the factors that may influence the availability and accessibility of interventions increases the likelihood that participants engage with an intervention long enough to access its therapeutic potential, a concept described within the construct A Therapeutic Foundation.

#### 3.3.2 A Therapeutic Foundation

This construct describes the emotional experience that results from the interaction between CYP feeling safe, unconstrained by their LTC, and having the opportunity to develop “therapeutic” relationships with intervention deliverers and/or fellow intervention recipients.

The presence of familiar people (e.g., Ayres et al.; Dennison et al., 2010) and ensuring that there are rules encouraging appropriate behaviour (e.g., Brodeur, 2005; Desai et al., 2014) and protect the privacy and/or anonymity of CYPs (e.g., Nicholas et al., 2007; Stewart et al., 2013a) can support participants to feel secure within an intervention setting. This perception of safety can help CYP “escape” the stigma and worry associated with their LTC experienced during their daily lives (e.g., Muskat et al., 2016). This feeling of being unconstrained can be facilitated by activities that distract CYP from the pain or boredom associated with long hospital stays (O'Callaghan, Dun, Baron, & Barry, 2013;Docherty et al., 2013) or help them overcome physical limitations associated with their LTC (Gillard & Watts, 2013). CYP with stigmatized diagnoses such as HIV (e.g., Gillard & Allsop, 2016) or altered appearances (e.g., Bluebond‐Langer et al., 1991; Tiemens et al., 2007) appear to particularly value this release from everyday worries or routine.

Feeling unconstrained within a safe space supports CYP to freely share their emotions and experiences (e.g., Fair et al., 2012). Given enough time, the unconstrained sharing of emotions and experiences can lead to the development of supportive “Therapeutic Relationships” between CYP taking part in the intervention (e.g., Tiemens et al., 2007). These relationships seem to be characterized by CYP feeling understood, cared for, and valued (e.g., Brodeur, 2005; Fair et al., 2012; et al., 2011). Together, the concepts described above can create a therapeutic atmosphere, which we suggest could enhance the well‐being of CYP with LTCs by itself. This ‘Therapeutic Foundation' also creates the potential for those receiving the intervention to engage with the social support offered by peers and experience feelings of hope and empowerment.

#### 3.3.3 Social Support

This construct conceptualizes ‘Social Support' as the sense of belonging experienced by CYP as they meet other peers with an LTC and the opportunity to learn new skills and improve relationships with healthy peers and family outside of the intervention setting.

Sharing experiences with intervention deliverers, peers, and/or family within a safe environment allows CYP to receive reassurance that they are not alone living with the difficulties they are experiencing (e.g., Campbell et al., 2010; Gan et al., 2010). CYP described experiencing powerful feelings of belonging within interventions that allowed them to meet peers similar to themselves (e.g., Gillard & Allsop, 2016). This experience of belonging can further contribute to the therapeutic atmosphere described within ‘A Therapeutic Foundation'.

Meeting similar peers also helps CYP learn how to replicate the empathetic, understanding, and validating relationships they valued as part of an intervention and improve their relationships with others outside of the intervention setting (e.g., Bignall, Luberto, Cornette, Haj‐Hamed, & Cotton, 2015; Desai et al., 2014). This can be achieved directly by involving family with the intervention (e.g., Gan et al., 2010) or indirectly through CYP applying the skills they learned outside of the intervention setting (e.g., Hosek et al., 2012; Stewart, Barnfather, Magill‐Evans, Ray, & Letourneau, 2011; Stewart, Masuda, Letourneau, Anderson, & McGhan, 2011). The type of social support most appreciated by CYP with LTCs may vary according to their individual preference, age, LTC, and developmental level. The need for contact with similar peers may increase as CYP become more aware of their social surroundings and encounter the typical challenges associated with this developmental stage.

Over the course of the intervention, there seems to be a transition from building therapeutic relationships with peers and staff within an intervention setting to replicating these types of relationships within the CYPs everyday family and social systems. This process may be facilitated by the feelings of hope and empowerment generated through participation in an intervention, as detailed within A Hopeful Alternative and Empowerment below.

#### 3.3.4 A Hopeful Alternative

This construct describes how meeting peers and taking part in everyday activities can help CYP develop a sense of hope for the future and an identity not solely focused on their LTC.

CYP described how having the opportunity to meet other CYP who are currently managing the challenges of living with their LTC (e.g., Barnetz & Feigin, 2012;White, 2014) can result in a realization that it is possible to live an enjoyable life despite the presence of an LTC. This may in turn motivate them to engage with others participating in the intervention and both teach and learn new skills to manage their physical and mental health.

CYP appear to value recognition that they are able to support others (e.g., Desai et al., 2014; Nicholas et al., 2007; Tiemens et al., 2007) and not merely the passive recipients of help. This realization may enable participant to offer support to others as depicted under A Therapeutic Foundation and Social Support. Thus, taking part in an intervention can be experienced as a collaborative process, where everyone brings expertise.

Consideration of the family and social systems that CYP belong to within the intervention (as discussed within Getting In and Staying In) may support the recognition that the child is more than their physical illness. Integrating aspects of daily life into interventions, such as completion of everyday chores, hobbies, and non‐LTC‐orientated conversation with family members (e.g., Dennison et al., 2010; Nicholas et al., 2007), appeared to aid CYP's realization that they could be “normal” despite their LTC. However, the needs of CYP who require end‐of‐life care may be different and should be met accordingly.

It appears having the opportunity to observe peers successfully managing life with an LTC, take part in everyday activities, and contribute meaningfully to their relationships with others can help CYP visualize a hopeful future for themselves, which can have a positive influence in their mental health.

#### 3.3.5 Empowerment

The interlinked and self‐reinforcing relationship between a young person's increased sense of their own ability to manage themselves and increased self‐esteem are conceptualized here as Empowerment.

Social support can help CYP acquire knowledge and skills regarding how to manage their physical, emotional, and social difficulties (Marsac et al., 2012; Stewart et al., 2013a). As they master these skills, CYP develop a sense of confidence in their own abilities to manage their future physical (e.g., Barnetz & Feigin, 2012) and mental (e.g., Jaser, Patel, Linsky, & Whittemore, 2014) health needs. There appears to be an interaction between feelings of increased hope and empowerment. Feeling empowered to manage their own well‐being may provide CYP with increased hope for their future. In turn, the experience of successfully completing fun, challenging and/or normative activities not associated with their LTC (e.g., Moola, Faulkner, White, & Kirsh, 2015) can lead to feelings of empowerment and increased self‐esteem. We suggest there needs to be a balance between participating in activities that challenge them, with the need for these activities to be tailored to their developmental and health needs. This balance will ensure that CYP with LTCs can experience success and feel empowered.

Feelings of empowerment, hope, and being valued within an intervention's therapeutic relationships can have a positive impact on CYPs long‐term mental health and their ability to access ongoing social support from family and peers both during an intervention and after it has ended.

## 4 DISCUSSION

Synthesis of first‐ and second‐order construct data across the 60 articles in this review yielded five overarching constructs: ‘Getting In and Staying In, Therapeutic Foundation, Social Support, A Hopeful Alternative, and Empowerment.' The line of argument presented in Figure 2 explores the relationship between these five constructs. It highlights how the social support received from service professionals, family members, and peers was highly valued by CYP with LTCs. Social support can facilitate CYP's initial engagement with an intervention, the development of therapeutic relationships, and sense of belonging, as well as facilitate the learning of skills to manage their physical and mental health. This is consistent with the social support model proposed by Stewart et al. (2013a) and Stewart, Letourneau, Masuda, Anderson, and McGhan (2013b), which differentiates between affirmational, emotional, and informational support. Gaysynsky, Romansky‐Poulin, and Arpadi (2015) propose the concept of “esteem support,” which encompasses “validation” and “compliment,” ideas included within the constructs ‘Therapeutic Foundation' and ‘Hope and Inspiration' emerging from this review. Health and social care staff play a key role in identifying and signposting of CYP and their families towards age‐appropriate social support. Recommendations to increase the mental health support for young people in schools and community health services (Joint Commissioning Panel for Mental Health, 2013) may provide an opportunity for services that support CYP with LTCs to utilize a more holistic approach and encourage CYP to develop their social connections with peers.

The value of hope was a particularly striking finding of this review, complementing a review by Venning et al. (2008), which highlighted how young people feel their chronic illness restricts their participation in normal life and the importance of developing a sense of hope. Our findings suggest the development of hope and acceptance can be facilitated by CYP with LTCs having the opportunity to meet peers with an LTC, improve relationships with healthy peers, and incorporating fun or challenging activities unrelated to their LTC into their daily lives. This may then contribute towards building an identity not centred on their LTC.

### 4.1 Strengths and limitations

This review was conducted according to best practice guidelines (CRD, 2009). The purposive sampling procedure described within the methods section meant each of the 60 articles included in this review contributed towards the final synthesis. Although the large number of studies and variety of interventions included in the review may have made it harder to report nuances within the data, each of the five overarching constructs was informed by a large number of articles, with at least 10 of these receiving a “high”‐quality rating. We relied upon the self‐report and reflexivity of the authors of primary studies to identify how their ideological approach influenced their results, which may have influenced our findings.

The constructs identified within this synthesis may represent core therapeutic components common across different types of intervention; however, we cannot assume these are applicable across all interventions and all CYP with any LTC. A more focused review would be required for clinicians wishing to understand experiences of/attitudes towards a specific intervention or views in relation to specific psychiatric diagnoses. The absence of data regarding participant's experiences of mental health difficulties means we do not know how the findings of this review can be applied to CYP with different mental health needs: from those with a diagnosed mental health disorder to those who may need support to maintain their mental well‐being.

### 4.2 Recommendations for future research and practise

This reviews findings review suggest that interventions to assist CYP with an LTC to improve their social support networks, develop a sense of hope, and feel empowered may improve their mental health and well‐being. The five constructs identified by this review offer a potential framework for health and social care professionals to consider when developing or refining existing interventions to improve and/or maintain the mental health of CYP with LTCs. In their linked‐evidence synthesis, Moore et al. (2019 ) highlight the paucity of research evaluating the effectiveness of the type of interventions included within this review, as only four of the included studies conducted their qualitative evaluation of an intervention as part of a randomized controlled trial (Barry, O'Callaghan, Wheeler, & Grocke, 2010; Bignall et al., 2015; Dennison et al., 2010; Jaser et al., 2014). Further randomized controlled trials are needed to evaluate whether interventions including the components/processes identified by this review are more effective at improving the mental health of CYP with LTCs than those which do not. These should be carried out alongside process evaluations to ensure the experience and practicalities of implementing these types of an intervention within a U.K. setting are fully understood.

## 5 CONCLUSION

This review explored the attitudes and experiences of CYP, caregivers, and practitioners towards interventions aiming to improve the mental health of CYP with LTCs. The synthesis elicited five key constructs that suggest important areas to consider during the development and delivery of mental health interventions. Future research should implement mixed methods approaches to evaluate such interventions in light of components identified by this research with CYP, in order to better understand what influences the success of interventions in CYP with different physical and mental health needs.

## Supporting information

Table S1: Key Characteristics of Included Studies

Table S2: Description of Interventions

Table S3: Quality Appraisal of Included Studies

Table S4: Articles which contribute towards each overarching construct and subtheme

## APPENDIX A EXAMPLE SEARCH STRATEGY WITHIN THE PSYCINFO (VIA OVIDSP) DATABASE

**Table:**

#	Searches	Results
1	Chronic Disease/	0
2	Neoplasms/	28,712
3	Diabetes Mellitus/	4,470
4	Asthma/	4,034
5	exp Respiratory Tract Diseases/	0
6	Cystic Fibrosis/	732
7	Cerebral Palsy/	4,202
8	exp Epilepsy/	22,725
9	exp Muscular Diseases/	0
10	exp Endocrine System Diseases/	0
11	exp Immune System Diseases/	0
12	exp HIV Infections/	0
13	exp Cardiovascular Diseases/	0
14	exp Nervous System Diseases/	0
15	exp Skin Diseases/	0
16	exp Digestive System Diseases/	0
17	exp Hematologic Diseases/	0
18	exp Otorhinolaryngologic Diseases/	0
19	exp Stomatognathic Diseases/	0
20	exp Eye Diseases/	0
21	exp Pain/	47,653
22	Disabled Children/	0
23	((chronic* or longterm or long‐term or “long standing” or physical) adj2 (condition* or ill* or disease* or disorder* or syndrome*)).ti,ab.	45,152
24	(cancer* or neoplas* or tumor* or tumour* or malignan* or carcinoma* or “bone marrow” or leukaemia or leukemia).ti,ab.	63,603
25	diabet*.ti,ab.	23,493
26	asthma*.ti,ab.	6,465
27	(respiratory adj2 (illness* or disease* or condition*)).ti,ab.	1,201
28	cystic fibrosis.ti,ab.	993
29	cerebral palsy.ti,ab.	5,460
30	quadriplegi*.ti,ab.	491
31	tetraplegi*.ti,ab.	291
32	diplegi*.ti,ab.	378
33	spinal cord injur*.ti,ab.	4,595
34	muscular dystrophy.ti,ab.	1,123
35	epilep*.ti,ab.	33,297
36	seizure*.ti,ab.	25,791
37	spina bifida.ti,ab.	868
38	(heart adj2 (condition* or disease* or disorder* or defect*)).ti,ab.	9,188
39	(cardiac adj2 (condition* or disease* or disorder* or defect*)).ti,ab.	1,390
40	(cardiovascular adj2 (condition* or disease* or disorder* or defect*)).ti,ab.	8,891
41	(skin adj2 (condition* or disease* or disorder*)).ti,ab.	966
42	eczema.ti,ab.	330
43	(allergies or allergy).ti,ab.	1,529
44	dermatitis.ti,ab.	532
45	(gastrointestinal adj (disorder* or disease*)).ti,ab.	669
46	((stomach or abdominal or gastrointestinal) adj pain).ti,ab.	1,342
47	(bowel* adj2 inflammatory adj2 (condition* or disease* or illness*)).ti,ab.	602
48	(liver adj (disease* or transplant*)).ti,ab.	1,621
49	hepatitis.ti,ab.	3,515
50	(disabilit* adj5 child*).ti,ab.	13,184
51	(human immunodeficiency virus or HIV).ti,ab.	42,006
52	AIDS.ti,ab.	30,925
53	(hyperthyroidism or hypothyroidism).ti,ab.	1,635
54	an?emia.ti,ab.	1,617
55	h?emophilia.ti,ab.	368
56	sickle.ti,ab.	1,162
57	((renal or kidney) adj (disease* or disorder*)).ti,ab.	1,900
58	nephrotic syndrome.ti,ab.	55
59	encephalomyelitis.ti,ab.	1,604
60	chronic fatigue syndrome.ti,ab.	1,946
61	((cleft or palate) adj lip).ti,ab.	295
62	craniofacial.ti,ab.	527
63	(deaf or deafness).ti,ab.	13,257
64	(hearing adj (defect* or disorder*)).ti,ab.	347
65	blindness.ti,ab.	5,420
66	((vision or visually or visual) adj (impaired or impairment*)).ti,ab.	4,261
67	((persistent or chronic or recurring or frequent) adj (headache* or migraine*)).ti,ab.	1,720
68	chronic pain.ti,ab.	11,547
69	fibromyalgia.ti,ab.	2,541
70	medically unexplained symptoms.ti,ab.	452
71	(spinal adj injur*).ti,ab.	301
72	or/1–71	335,060
73	(child or children*).ti,ab.	529,036
74	(adolescent or adolescents).ti,ab.	169,875
75	teen*.ti,ab.	18,103
76	(young adj (adult* or people)).ti,ab.	53,922
77	youth*.ti,ab.	73,029
78	73 or 74 or 75 or 76 or 77	701,901
79	Mental Health/	47,847
80	(psychological adj (illness* or disorder* or difficulties or problems or distress)).ti,ab.	23,105
81	(mental adj (health or illness* or disorder* or distress or problem*)).ti,ab.	186,832
82	(depression or depressed or depressive).ti,ab.	235,411
83	((disruptive or challenging or antisocial) adj behavio?r).ti,ab.	11,412
84	(behavio?r adj problem*).ti,ab.	12,771
85	(anxiety or anxious).ti,ab.	157,623
86	feelings.ti,ab.	57,562
87	Internali*.ti,ab.	3
88	(wellbeing or well being).ti,ab.	61,891
89	happiness.ti,ab.	11,767
90	worry.ti,ab.	7,030
91	distress.ti,ab.	48,777
92	satisfaction.ti,ab.	82,607
93	emotional.ti,ab.	174,569
94	coping.ti,ab.	61,721
95	or/79–94	828,319
96	qualitative research/	6,724
97	Ethnology/	1,943
98	exp Questionnaires/	15,996
99	phenomenology/	11,126
100	Attitudes/	23,102
101	interviewing/	3,181
102	interview*.ti,ab.	250,183
103	qualitative.ti,ab.	110,757
104	(talked or asked).ti,ab.	81,112
105	focus group*.ti,ab.	24,246
106	ethnograph*.ti,ab.	20,873
107	grounded theory.ti,ab.	11,222
108	thematic.ti,ab.	14,905
109	(barriers and (facilitators or enablers)).ti,ab.	2,521
110	process evaluation.ti,ab.	1,094
111	group discussion*.ti,ab.	7,342
112	perception*.ti,ab.	232,038
113	attitude*.ti,ab.	171,590
114	views.ti,ab.	56,281
115	experience*.ti,ab.	486,970
116	or/96–115	1,107,429
117	intervention.ti,ab.	171,181
118	psychotherapy.ti,ab.	77,346
119	(support adj3 (group* or network)).ti,ab.	13,125
120	therapy.ti,ab.	194,016
121	counselling.ti,ab.	9,299
122	peer support.ti,ab.	2,673
123	social support.ti,ab.	35,639
124	program*.ti,ab.	318,905
125	(mental adj3 service*).ti,ab.	22,848
126	training.ti,ab.	210,131
127	technique*.ti,ab.	158,051
128	treatment*.ti,ab.	508,051
129	or/117–128	1,226,579
130	72 and 78 and 95 and 116 and 129	4,011

## APPENDIX B LIST OF WEBSITES SEARCHED

Mental Health Foundation https://www.mentalhealth.org.uk/

Young Minds http://www.youngminds.org.uk/

Department of Child and Adolescent Mental Health Service (CAMHS) at Great Ormond Street Hospital http://www.gosh.nhs.uk/medical-information/clinical-specialties/child-and-adolescent-mental-health-services-camhs-information-parents-and-visitors/research-and-publications

Child & Adolescent Mental Health Services (CAMHS) Research Unit http://www.cpcs.org.uk/index.php?page=high-needs-project

NIHR CRN: Children and young people's mental health research https://www.crn.nihr.ac.uk/mentalhealth/about-mental-health-research/children-and-young-peoples-mental-health-research/

NIMH Child and Adolescent Mental Health http://www.nimh.nih.gov/health/topics/child-and-adolescent-mental-health/index.shtml#part_152583

RCP CAMHS Resource Library http://www.rcpsych.ac.uk/quality/qualityandaccreditation/childandadolescent/communitycamhsqncc/camhsresourcelibrary.aspx

National Child and Maternal Health Intelligence Network http://www.chimat.org.uk/camhs

National Children's Bureau http://www.ncb.org.uk/healthy-care/useful-links/mental-health-and-emotional-well-being

Association for Children's Mental Health http://www.acmh-mi.org/get-information/

Substance Abuse and Mental Health Services Administration http://www.samhsa.gov/data/

American Academy of Pediatrics https://www.aap.org/en-us/professional-resources/Research/Pages/Research.aspx

Child and Youth Health http://www.cyh.com/HealthTopics/HealthTopicDetails.aspx

The Kings Fund: Mental Health http://www.kingsfund.org.uk/topics/mental-health

Young Epilepsy: http://www.youngepilepsy.org.uk/

Epilepsy Society: https://www.epilepsysociety.org.uk/young-people-and-epilepsy#.Vz8FjuTAN_A

Epilepsy Action: https://www.epilepsy.org.uk/info/young-people

Cancer Research UK: http://www.cancerresearchuk.org/health-professional/cancer-statistics/teenagers-and-young-adults-cancers

Macmillan: http://www.macmillan.org.uk/cancerinformation/teensandyoungadults/infoforteensandyoungadults.aspx

Teenage Cancer Trust: https://www.teenagecancertrust.org/get-help/ive-got-cancer/stories-others

Teenagers and Young Adults with Cancer: http://www.tyac.org.uk/

Sickle Cell and young stroke survivors: http://www.scyss.org/

Sickle Cell Society: http://sicklecellsociety.org/resources/did-you-know-age-11-16-an-information-booklet-for-young-people/

AVERT (HIV): http://www.avert.org/living-with-hiv/health-wellbeing/being-young-positive

Young people with Aids: http://www.amfar.org/about-hiv-and-aids/young-people-and-hiv/young-people-and-hiv-aids/

Pozitude: http://www.pozitude.co.uk/

Children and Young People HIV network: http://www.ncb.org.uk/hiv

Child's Brain Injury Trust: http://childbraininjurytrust.org.uk/how-we-help/young-people/

Stroke Association: https://www.stroke.org.uk/finding-support/information-about-stroke-childhood

National Institute of Neurological Disorders and Stroke: http://www.ninds.nih.gov/disorders/tbi/detail_tbi.htm

Cerbera: http://w3.cerebra.org.uk/

Association of young people with M.E.: http://www.ayme.org.uk/

Away with Pain: http://www.awaywithpain.co.uk/young-people

Fibromyalgia: http://www.fmauk.org/information-packs-mainmenu-58/booklet-mainmenu-135/232-children-and-young-people

http://www.fmscommunity.org/pediatric.htm

Healing Well: http://www.healingwell.com/community/default.aspx?f=24&m=1813258

Arthritis research UK: http://www.arthritisresearchuk.org/arthritis-information/young-people.aspx

Youth Health: http://www.healthtalk.org/young-peoples-experiences/arthritis/what-arthritis-and-what-are-early-symptoms

Arthritis Care: https://www.arthritiscare.org.uk/managing-arthritis/children-with-arthritis

CLIC Sargent http://www.clicsargent.org.uk/content/help-and-support

Cerebral Palsy http://www.cerebralpalsy.org/information/child-care

Asthma UK https://www.asthma.org.uk/research/

British Lung Foundation https://www.blf.org.uk/what-we-do/research

Diabetes UK https://www.diabetes.org.uk/Research/

British Deaf Association https://www.bda.org.uk/publications

Mental Elf http://www.nationalelfservice.net/

## APPENDIX C DESCRIPTION OF HOW ARTICLES MEETING THE INCLUSION CRITERIA FOR THE REVIEW WERE ENTERED INTO THE RECIPROCAL TRANSLATION PROCESS

Abbreviations: CYP, children and young people; MH, mental health.

*Articles that reported views of multiple population groups but synthesized the views of CYP, parent, and/or practitioner views separately were included in multiple groups within this synthesis. Thus, total number of articles included across different groups exceeds 20. ^†^See Table S1 for details regarding at which stage each included article entered the reciprocal translation process.
