# Supplementary material for: Experiences of interventions aiming to improve the mental health and well‐being of children and young people with a long‐term physical condition: A systematic review and meta‐ethnography
Source: Child Care Health Dev. 2019 Aug 16;45(6):832–49. doi: 10.1111/cch.12708 (PMC6851835; doi:10.1111/cch.12708)
Supplement: Supplementary file 1 — Table S1: Key Characteristics of Included Studies [file CCH-45-832-s001.docx]

Table S1: Key Characteristics of Included Studies

| **First author (Date)** | **Country** | **Publication type** | **Study focus** | **Intervention type** | **Qualitative data collection method** | **Total sample size (N)** | **Details of participants providing qualitative data (N, who, % female, mean age years (SD)** | **Type of qualitative data analysis** | **Stage article entered reciprocal translation process (1, 2, 3)** |
| --- | --- | --- | --- | --- | --- | --- | --- | --- | --- |
| [Ayers (2011](#_ENREF_1)) | United Kingdom | JAP | Views about interventions received | Inhaled nitrous oxide, Play therapy, Parent support | Semi-structured interviews | 28 | 14 White-European CYP with **Cystic Fibrosis**, 64.3% f, 12.4 years (range 7-15), 14 Parents, 78.6 f, 41.5 years (NR) | Systematic thematic analysis | 2 |
| [Barlow (1999](#_ENREF_2)) | United Kingdom | JAP | Views on intervention in development | Psycho-educational | Five focus groups and two individual interviews | 30 | 10 CYP with **Juvenile Arthritis**, 50 f, (range 8-15 years); 13 Parents, 69.2% f ; 7 Health professionals | Framework analysis | 1 |
| [Barnetz (2012](#_ENREF_3)) | Israel | JAP | Views about intervention received | Mentoring | Semi-structured interviews, documented in writing during the interview. | 24 | 24 CYP with **Type 1 Diabetes**, 46.9% f, 14 years (2.36) | Inductive content analysis based on grounded theory | 1 |
| [Barnfather (2011](#_ENREF_4));  [Stewart (2011](#_ENREF_50)b) | Canada | JAP | Mixed methods intervention evaluation | Online peer support (with Peer Mentors) | Online discussion transcripts; peer mentor field notes and interviews; CYP interviews | 27 | 22 CYP with LTC (50% **Cerebral Palsy**, 50% **Spina Bifida**) , 45.5% f, 14.6 years (1.6); 5 peer mentors (40% Cerebral Palsy, 60% Spina Bifida) 60 f | Inductive thematic analysis | Barnfather (2011): 3 Stewart (2011b): 1 |
| [Barry (2010](#_ENREF_5)) | Australia | JAP | RCT evaluating intervention with qualitative component | Music therapy | CYP interviews, parent/staff questionnaires, therapist-researcher reflexive journal | 45 | 11 CYP with **Cancer**, 50% f, Median age: 8 years (median); 11 Parents; 1 Music Therapy Researcher; 11 Treatment Staff; 11 Planning Staff | Thematic analysis based on modified grounded theory process | 2 |
| [Baruch, (2010](#_ENREF_6)) | USA | D | Views about intervention received | Narrative/ Psychosocial support/Reward programme | Interviews, focus groups and surveys | 29 | 6 CYP with **Cancer**, NR, 12.7 years (range 10-17); 8 Parents, 75% f, 36 years (range 31-45); 9 Practitioners; 6 Bead artists, NR, 21 years+ | Qualitative descriptive methods: Inductive content analysis. | 2 |
| [Bignall (2015](#_ENREF_8)) | USA | JAP | RCT evaluating intervention with qualitative component | Relaxation (Breathing) | Interviews after visits one and two. | 30 | 15 African American CYP with **Asthma**, 66.7% f, 15.53 years (1.5) | Thematic analysis | 2 |
| [Bluebond-Langer (1991](#_ENREF_9)) | USA | JAP | Views about intervention received. | Camp | CYP interviews, parent questionnaires and observations | 50 | 50 CYP with **Cancer**, 42% f, 12 years (range 7-16 years) | Ethnography. Each transcript was independently coded (code book designed by the authors) | 1 |
| [Brodeur (2005](#_ENREF_10)) | USA | D | Views about intervention received | Family art therapy | Semi-structured interviews | 25 | 25 Total, 56.7% f, range 4-45 years 9 CYP with LTC; 13 Parents (9 mothers, 4 fathers); 3 Siblings | Interpretative phenomenolog-ical analysis | 1 |
| [Brothers (2014](#_ENREF_11)) | USA | JAP | Views about intervention received | Group intervention/ Group therapy | Two open-ended items [from Session evaluation forms], three intervention deliverer interviews | 25 | 22 female CYP with **HIV**, 0.55 years (16-24 years); 3 Intervention deliverers | Thematic analysis | 3 |
| [Bultas (2015](#_ENREF_12)) | USA | JAP | Mixed methods intervention evaluation | Camp | CYP Photovoice interviews; parent open-ended survey questions. | 50 | 50 CYP with **CHD**-Parent dyads, NR, range 8-15 years | Qualitative description. Non abstract/ interpretive, low inference, straight forward description of phenomena | 1 |
| [Burns (2010](#_ENREF_13)) | USA | JAP | Views about intervention received | Music therapy | Interview | 7 | 7 Parents of CYP with **Cancer**, 57.14% f, 16 years (range 13-21 years) | Colaizzi’s empirical phenomenology-ical approach | 2 |
| [Campbell (2010](#_ENREF_14)) | United Kingdom | JAP | Views about intervention received | Group work | Interviews (telephone or in person during clinic appointment) | 6 | 6 African Adolescents with **HIV**, 50% f, range 13-15 years | Thematic analysis | 2 |
| [Curle (2005](#_ENREF_16)) | United Kingdom | JAP | Views about intervention received | Group therapy | Interviews semi-structured | 27 | 11 White Children with LTC, 45.5% f, range 7-12 years; 14 Parents, 71.4% f, NR, 2 Siblings, 50 f, NR | Grounded Theory | 2 |
| [Dennison (2010](#_ENREF_17)) | United Kingdom | JAP | RCT evaluating intervention with qualitative component | CBT, psycho-education | Telephone interviews | 32 | 16 White-British CYP with **CFS**, 62.5% f, 19.9 years (range 16-24); 16 Parents, 87.5 f, NR | Inductive thematic analysis | 1 |
| [Desai (2014](#_ENREF_18)) | USA | JAP | Views about intervention received | Camp | Participant generated photography and semi-structured interviews | 13 | 13 CYP with **CHD**, 38.46% f, 12.7 years (range 9-16) | Thematic analysis: Highlighting approach | 1 |
| [Docherty (2013](#_ENREF_19)) | USA | JAP | Views about intervention received | Music therapy | Unstructured qualitative interviews | 16 | 16 Parents of CYP with **Cance**r, 87.5% f, 44.5 years (range 30-51) | Adaptation of Colaizzi’s Phenomenolog-ical method of analysis | 2 |
| [Fair (2012](#_ENREF_20)) | USA | JAP | Views about intervention received | Creative writing (Group) | Interviews | 32 | 7 Adolescents with **HIV**, NR, 16 years (range 13-20); 4 Carers, 50% f, 55.3 years (range 45-71); 5 Group leaders, NR, 32 years (range 26-50) | Grounded Theory | 1 |
| Gan (2010) | USA | JAP | Views about intervention received | Family intervention/ therapy | Questionnaires and semi-structured interviews with family members and clinicians. | 25 | 8 Adolescents with **ABI**, 37.5% f, 14.6 years (2.1); 14 Family members (9 parents, 1 partner, 4 siblings); 3 Clinicians, NR details | Content analysis | 2 |
| [Gaysynsky (2015](#_ENREF_21)) | USA | JAP | Views about intervention received | Online support group | Online posts and comments transcripts from March 1^st^ 2011 to July 1^st^ 2012 | 43 | 43 CYP with **HIV**, 34.9% f, range 18-27 years. | Directed content analysis | 3 |
| [Gillard (2011](#_ENREF_24)) | USA | JAP | Views about intervention received | Camp | In 2007: Camper focus groups, staff interviews and informal observations, interviews with former campers. In 2008: Formal and informal interviews with campers and staff, participant observations, and artefact reviews. | 51 | 36 CYP with **HIV**, NR, range 7-19 years; 15 Staff, Other details NR | Grounded Theory as outlined by Strauss and Corbin (1998). Analysis using the constant comparison method | 1 |
| [Gillard (2013](#_ENREF_23)) | USA | JAP | Views about intervention received | Camp | Semi-structured interviews with campers and junior counsellors, observations. Focus groups with staff and 12 campers. | 24 | 10 CYP with **Cancer**, NR, range 12-14 years; 4 Junior Counsellors, NR, range 18-20 years; 10 Staff | Constant comparison method | 1 |
| [Gillard (2016](#_ENREF_22)) | USA | JAP | Views about intervention received | Camp | Video interviews | 24 | 24 CYP with LTC, 70.83% f, 15 years (0.46). | Interpretative phenomenolo-gical analysis | 1 |
| [Griffiths (2015](#_ENREF_25)) | United Kingdom | JAP | Views about intervention before and after received | Online support group | Pre-intervention focus groups. Views posted in forum during intervention. Post-intervention focus groups.  Analysis of forum messages | 12 | 12 CYP with **Cancer**, 58.30% f, 21.08 years (4.15) | Inductive thematic analysis | 3 |
| [Hosek (2012](#_ENREF_26)) | USA | JAP | Views about intervention in development | Secondary prevention | Focus groups | 17 | 17 female CYP with **HIV**, 21 years (range 17-24) | Thematic analysis | 1 |
| [Jaser (2014](#_ENREF_27)) | USA | JAP | RCT evaluating intervention with qualitative component | Positive affirmation | Semi-structured interviews | 37 | 20 Adolescents with **Type 1 Diabetes**, 60% f, 15.3 years (1.4); 17 Parents, 83.3 f | Content analysis | 2 |
| [Kashikar-Zuck (2016](#_ENREF_28)) | USA | JAP | Mixed methods intervention evaluation | CBT + Neuro-muscular exercise training | Interview | 17 | 17 Adolescents with **JFM**, 100% f, 16(2.15) | Thematic analysis | 2 |
| [Kirk (2016](#_ENREF_29)) | United Kingdom | JAP | Views about intervention received | Online support group | Website posts, Observational notes | 279 | 97 CYP with **CF**; 182 Parents | Inductive grounded theory | 2 |
| [Lewis (2016](#_ENREF_30)) | Australia | JAP | Mixed methods intervention evaluation | Peer support | Focus groups, One-to-one interviews with coordinators | 32 | 4 Adolescents with LTC (new members), 100% f, 12-17 years; 4 Adolescents with LTC (established members), 50% f, 17-23 years; 19 Parents of new members, 84.2% f; 5 Co-ordinators, 100 f | Thematic analysis | 1 |
| [MacDonald (2010](#_ENREF_31)) | United Kingdom | JAP | Views about intervention received | Befriending programme | Semi-structured interviews, focus group | 14 | 5 CYP with **Cystic Fibrosis**, NR, 13.4(range 8-18); 5 Parents; 4 Practitioners; 3 Befrienders | ‘Framework’ model was used to build a matrix of themes and codes from the four sets of data | 3 |
| [Marsac (2012](#_ENREF_32)) | USA | JAP | Views about intervention before and after received | Coping strategies | Semi-structured and cognitive interviews | 30 | 15 CYP with **Cancer**, 46.7% f, 8.8 years (1.7); 15 Parents, 80 f, NR | Thematic analysis assumed | 2 |
| [Masuda (2013](#_ENREF_33)) | Canada | JAP | Views about intervention before and after received | Online support | Individual interviews (face to face, telephone), group interview, open-ended survey questions | 27 | 14 CYP with **Asthma and Life threatening allergies**, NR, 12-15 years  8 parents 5 peer mentors | Framework analysis | 2 |
| [Moola (2015](#_ENREF_35)) | Canada | JAP | Views about intervention received | Camp | Semi-structured interviews | 15 | 15 CYP with **CHD**, 60% f, range 9-16 years | Interpretative phenomenological analysis | 1 |
| [Muskat (2016](#_ENREF_36)) | Canada | JAP | Views about intervention received | Support group | Semi-structured interviews | 25 | 16 CYP with **HIV**, 37.5% f, Range 11-18 years; 9 Caregivers, 88.9 f, NR | McCracken’s “Long Interview” method of qualitative data analysis | 1 |
| [Nicholas (2007](#_ENREF_37)) | Canada | JAP | Views about intervention received | Online games, education and peer support network | Ethnographic semi-structured interviews | 19 | 9 CYP with LTC, 22.2% f, 11.3 years (3.42); 7 family caregivers; 3 Health care providers | Interviews were audio-recorded, transcribed verbatim and subjected to theme generation | 1 |
| [Nicholas (2009](#_ENREF_39)) | Canada | JAP | Mixed methods intervention evaluation | Asthma education delivered at summer camp | Semi-structured interviews | 22 | 22 CYP with **Asthma**, 31.81% f, range 7-15 years | Transcripts were subjected to code identification, category development, and theme generation | 3 |
| [Nicholas (2012](#_ENREF_38)) | Canada | JAP | Mixed methods intervention evaluation | Online education and support | Interviews: Long interview approach | 15 | 15 CYP with **Type 1 diabetes** in intervention group, 14.5% f, range 12-17 years | Transcripts were content analysed and themes were generated | 3 |
| [Nieto (2015](#_ENREF_40)) | Spain | JAP | Views about intervention before and after received | Online psychoeducation | Semi-structured, family interviews (face-to-face or by video conference) | 15 | 15 CYP with **FAP**, 60, Median age 11(range 9-14) and their Parents | Inductive content analysis | 2 |
| [Nilsson (2009](#_ENREF_41)) | Sweden | JAP | Mixed methods intervention evaluation | Game | Semi-structured interviews | 21 | 21 CYP with **Cancer**, 33.3% f, median age 11 years (range 5-18) | Content analysis. The meaning units were subsequently abstracted to categories and eventually abstracted to themes | 2 |
| [O'Callaghan (2011](#_ENREF_42)) | Australia | JAP | Views about intervention received | Music/Music therapy | Semi-structured interviews | 54 | 26 CYP with **Cancer**, 34.5% f, median (25th-75th percentile) 7.5 years (range 4.6-8.2); 28 Parents, 82.1 f, NR | Grounded theory:  Comparative analytical process | 2 |
| [O'Callaghan (2012](#_ENREF_43)) | Australia | JAP | Views about intervention received | Music therapy | Semi-structured interviews | 12 | 12 CYP with **Cancer**, 58.33% f, 21 years (2.7) | Grounded theory and thematic analysis | 2 |
| [O'Callaghan (2013](#_ENREF_44)) | Australia | JAP | Views about interventions delivered | Music/Music therapy | Semi-structured focus groups, therapists’ written reflections | 4 | 4 female music therapists, NR | Grounded theory and thematic analysis | 2 |
| [Reme (2013](#_ENREF_45)) | United Kingdom | JAP | Mixed methods intervention evaluation | Lightening Process | Semi-structured telephone interviews | 12 | 9 CYP with **CFS**, 88.89% f, range 14-26 years; 3 Mothers, NR | Inductive thematic analysis | 2 |
| [Romero (2014](#_ENREF_46)) | USA | D | Views about intervention received | Online support, social networking and education Site | Posts on social networking (intervention) site | 74 | 74 CYP with **Cystic Fibrosis**, 71.6% f, 14.53 years (2.06) | Deductive thematic content analysis | 3 |
| [Serlachius (2012](#_ENREF_47)) | Australia | JAP | Views about intervention in development | Coping skills | Semi-structured focus groups | 13 | 13 Adolescents with **Type 1 Diabetes**, 30.77% f, 15.4 years (1.7) | Deductive thematic analysis | 2 |
| [Shrimpton (2013](#_ENREF_48)) | Australia | JAP | Views about intervention received | Creative therapy/ Narrative therapy | Semi-structured interviews | 10 | 40 Parents of CYP with **Cancer** | Thematic analysis | 2 |
| [Sibinga (2011](#_ENREF_49)) | USA | JAP | Mixed methods intervention evaluation | Mindfulness-based stress reduction | Interviews | 5 | 5 CYP with **HIV**, 80% f, 17 years (range 13-21) | Constant comparative method and content analysis | 2 |
| [Stewart (2011](#_ENREF_53)a) | Canada | JAP | Mixed methods intervention evaluation | Games and online peer support | Individual telephone interviews | 55 | 20 CYP with **Asthma and/or Severe Allergies**, NR, 8.64 years (1.06); 35 Parents | Constant comparative content analysis | 3 |
| [Stewart (2013](#_ENREF_52)a)  [Stewart 2013](#_ENREF_51)b) | Canada | JAP | Mixed methods intervention evaluation | Games and online peer support | Session recordings, participant emails, individual and group interviews | 57 | 27 CYP with **Asthma and Severe Allergies** (63%) or **Severe Allergies only**, NR, range 7-11 years; 25 Parents; 5 Peer Mentors | Thematic content analysis | Stewart (2013a): 1 Stewart (2013b): 3 |
| [Stinson (2008](#_ENREF_55)) | Canada | JAP | Views about intervention in development | Online self-management /education programme | Individual interviews and focus groups | 36 | 36 CYP with **JIA**, 66.7% f, 15.1 years (2.1) | Thematic analysis | 3 |
| [Stinson (2010](#_ENREF_54)) | Canada | JAP | Views about in development | Online support | Semi-structured interviews and observations | 38 | 19 CYP with **JIA**, 74% f, 15.7 years (1.5) 19 Parents, 63 f, 53% 40-49 years | Content analysis | 3 |
| [Tiemens (2007](#_ENREF_57)) | Canada | JAP | Mixed methods intervention evaluation | Camp | Focus groups | 7 | 4 CYP with **craniofacial difference**, 100% f, 16.2 years (14-18); 3 parents | Constant comparative method. | 1 |
| [Weekes (1993](#_ENREF_58)) | USA | JAP | Mixed methods intervention evaluation | Hand-holding | Observations and semi-structured interviews | 20 | 10 CYP with **Cancer**, 50% f, 15.7 years (range 12-18); 10 CYP with **Renal Failure**, 50% f, 14.8 years (range 11-19) | Constant comparative techniques from grounded theory methodology. | 2 |
| [White (2014](#_ENREF_59)) [White (2016](#_ENREF_60)) | Canada | D, JAP | Views about intervention received | Camp | Semi-structured interviews | 9 | 9 Parents of CYP with **CHD**, 88.9% f, 42 years (range 32-51) | Thematic analysis | White (2014): 2 White (2016): 3 |
| [Whittemore (2010](#_ENREF_61)) | USA | JAP | Views about intervention before and after received | Internet coping skills training program | Focus groups, think-aloud process, and the survey | 13 | 13 Adolescents with **Type 1 Diabetes**, 40% f, 14 years (1.15); NR Parents | Content analysis method | 2 |
| [Wolf Bordonaro (2005](#_ENREF_62)) | USA | D | Mixed methods intervention evaluation | Art therapy | Ethnographic research techniques | 3 | 3 female CYP with **SCD**, Range 6-9 years | Ethnographic analysis | 2 |
| [Wright (2004](#_ENREF_63)) | USA | JAP | Views about intervention received | Physical activity | Documentation of student progress structured interviews, case-specific questions, informal follow up interviews | 26 | 7 CYP with **Cerebral Palsy**, 14.3% f ,range 4-11 years; 4 female Practitioners, NR; 5 Parents, 80 f, NR | Inductive analysis and constant comparison | 1 |

^a^Intervention or components named in study, 1=Entered into first stage of synthesis, 2=Entered into second stage of synthesis (purposive sampling), 3=Entered into third stage of synthesis. BIFI-A = Brain Injury Family Intervention for Adolescents, CBT = Cognitive Behavioural Therapy, CFS = Chronic Fatigue Syndrome, CHD = Congenital Heart Disease, ChIPS = Chronic Illness Peer Support, D=Dissertation, DMAP = Developmental Martial Arts Program, FAP = Functional Abdominal Pain, HRQOL = Health Related Quality of Life, JAP=Peer reviewed journal article, JFM = Juvenile Fibromyalgia, MBSR = Mindfulness Based Stress Reduction, MTCD = Music Therapy CD Creation, NA = Not Applicable, NR = Not Reported, PSRM = Personal and Social Responsibility Model, TMV = Therapeutic Music Video, TTG = Terrific Tuesday Group, VR = Virtual Reality , YAP = Young Adults Programme , YMCA = Young Men’s Christian Association

## Bibliography of included studies

Ayers, S., Muller, I., Mahoney, L. & Seddon, P. (2011) Understanding needle-related distress in children with cystic fibrosis. *British Journal of Health Psychology, 16*(Pt 2), 329-343. DOI: http://dx.doi.org/10.1348/135910710X506895.

Barlow, J. H., Shaw, K. L. & Harrison, K. (1999) Consulting the 'experts': children's and parents' perceptions of psycho-educational interventions in the context of juvenile chronic arthritis. *Health Education Research, 14*, 597-610.

Barnetz, Z. & Feigin, R. (2012) "We didn't have to talk": Adolescent perception of mentor-mentee relationships in an evaluation study of a mentoring program for adolescents with juvenile diabetes. *Child & Adolescent Social Work Journal, 29*, 463-483. DOI: http://dx.doi.org/10.1007/s10560-012-0273-1.

Barnfather, A., Stewart, M., Magill-Evans, J., Ray, L. & Letourneau, N. (2011) Computer-mediated support for adolescents with cerebral palsy or spina bifida. *CIN: Computers, Informatics, Nursing, 29*, 24-35 12p. DOI: 10.1097/NCN.0b013e3181f9db63.

Barry, P., O'Callaghan, C., Wheeler, G. & Grocke, D. (2010) Music therapy CD creation for initial pediatric radiation therapy: A mixed methods analysis. *Journal of Music Therapy, 47*, 233-263.

Baruch, J. M. (2010). *The Beads of Courage program for children coping with cancer.* [Ph.D.], University of Arizona.

Bignall, W. J. R., Luberto, C. M., Cornette, A. F., Haj-Hamed, M. & Cotton, S. (2015) Breathing retraining for African-American adolescents with asthma: A pilot study of a school-based randomized controlled trial. *Journal of Asthma, 52*, 889-896. DOI: http://dx.doi.org/10.3109/02770903.2015.1033724.

Bluebond-Langer, M., Perkel, D. & Goertzel, T. (1991) Pediatric cancer patients' peer relationships: The impact of an oncology camp experience. *Journal of Psychosocial Oncology, 9*, 67-80.

Brodeur, S. S. (2005) Treating families coping with chronic illness: An evaluation of the "Living Well" program. *Dissertation Abstracts International: Section B: The Sciences and Engineering, 66*(5-B), 2811.

Brothers, J., Harper, G. W., Fernandez, M. & Hosek, S. G. (2014) EVOLUTION-Taking charge and growing stronger: The design, acceptability, and feasibility of a secondary prevention empowerment intervention for young women living with HIV. *AIDS Patient Care and STDS, 28*, 33-42. DOI: http://dx.doi.org/10.1089/apc.2013.0085.

Bultas, M. W., Schmuke, A. D., Moran, V. & Taylor, J. (2015) Psychosocial Outcomes of Participating in Pediatric Diabetes Camp. *Public Health Nursing*. doi: 10.1111/phn.12218

Burns, D. S., Robb, S. L., Phillips-Salimi, C. & Haase, J. E. (2010) Parental perspectives of an adolescent/young adult stem cell transplant and a music video intervention. *Cancer Nursing, 33*(4), E20-27. DOI: http://dx.doi.org/10.1097/NCC.0b013e3181d4b671.

Campbell, T., Beer, H., Wilkins, R., Sherlock, E., Merrett, A. & Griffiths, J. (2010) "I look forward. I feel insecure but I am ok with it". The experience of young HIV+ people attending transition preparation events: a qualitative investigation. *AIDS Care, 22*, 263-269. DOI: http://dx.doi.org/10.1080/09540120903111460.

Curle, C., Bradford, J., Thompson, J. & Cawthron, P. (2005) Users' views of a group therapy intervention for chronically Ill or disabled children and their parents: Towards a meaningful assessment of therapeutic effectiveness. *Clinical Child Psychology and Psychiatry, 10*, 509-527. DOI: http://dx.doi.org/10.1177/1359104505056315.

Dennison, L., Stanbrook, R., Moss-Morris, R., Yardley, L. & Chalder, T. (2010) Cognitive behavioural therapy and psycho-education for chronic fatigue syndrome in young people: reflections from the families' perspective. *British Journal of Health Psychology, 15*(Pt 1), 167-183. DOI: http://dx.doi.org/10.1348/135910709X440034.

Desai, P., Sutton, L., Staley, M. & Hannon, D. (2014) A qualitative study exploring the psychosocial value of weekend camping experiences for children and adolescents with complex heart defects. *Child: Care, Health and Development, 40*, 553-561. DOI: http://dx.doi.org/10.1111/cch.12056.

Docherty, S. L., Robb, S. L., Phillips-Salimi, C., Cherven, B., Stegenga, K., Hendricks-Ferguson, V., Roll, L., Donovan, S. M. & Haase, J. (2013) Parental Perspectives on a Behavioral Health Music Intervention for Adolescent/Young Adult Resilience During Cancer Treatment: Report From the Children's Oncology Group. *Journal of Adolescent Health, 52*, 170-178. DOI: 10.1016/j.jadohealth.2012.05.010.

Fair, C. D., Connor, L., Albright, J., Wise, E. & Jones, K. (2012) “I’m positive, I have something to say”: Assessing the impact of a creative writing group for adolescents living with HIV. *The Arts in Psychotherapy, 39*, 383-389. DOI: https://doi.org/10.1186/1472-6963-14-348.

Gan, C., Gargaro, J., Krutzer, J. S., Boschen, K. A. & Wright, F. V. (2010) Development and preliminary evaluation of a structured family system intervention for adolescents with brain injury and their families. *Brain Injury,* 24, 651-663. DOI: http://dx.doi.org/10.3109/02699051003692142.

Gaysynsky, A., Romansky-Poulin, K. & Arpadi, S. (2015) "My YAP Family'': Analysis of a Facebook Group for Young Adults Living with HIV. *AIDS and Behavior, 19*, 947-962. DOI: 10.1007/s10461-014-0887-8.

Gillard, A. & Allsop, J. (2016) Camp experiences in the lives of adolescents with serious illnesses. *Children and Youth Services Review, 65*, 112-119. doi: 10.1016/j.childyouth.2016.04.001

Gillard, A. & Watts, C. E. (2013) Program features and developmental experiences at a camp for youth with cancer. *Children and Youth Services Review, 35*, 890-898. DOI: http://dx.doi.org/10.1016/j.childyouth.2013.02.017.

Gillard, A., Witt, P. A. & Watts, C. E. (2011) Outcomes and processes at a camp for youth with HIV/AIDS. *Qualitative Health Research, 21*, 1508-1526. DOI: http://dx.doi.org/10.1177/1049732311413907.

Griffiths, C., Panteli, N., Brunton, D., Marder, B. & Williamson, H. (2015) Designing and evaluating the acceptability of Realshare: An online support community for teenagers and young adults with cancer. *Journal of Health Psychology, 20*, 1589-1601. DOI: 10.1177/1359105313519154.

Hosek, S., Brothers, J., Lemos, D. & the Adolescent Medicine Trials Network for HIV/AIDS Interventions (2012) What HIV-positive young women want from behavioral interventions: A qualitative approach. *AIDS Patient Care and STDS, 26*, 291-297. DOI: 10.1089/apc.2011.0035.

Jaser, S. S., Patel, N., Linsky, R. & Whittemore, R. (2014) Development of a positive psychology intervention to improve adherence in adolescents with type 1 diabetes. *Journal of Pediatric Health Care, 28*, 478-485. DOI: http://dx.doi.org/10.1016/j.pedhc.2014.02.008.

Kashikar-Zuck, S., Tran, S. T., Barnett, K., Bromberg, M. H., Strotman, D., Sil, S., Thomas, S. M., Joffe, N., Ting, T. V., Williams, S. E. & Myer, G. D. (2016) A Qualitative Examination of a New Combined Cognitive-Behavioral and Neuromuscular Training Intervention for Juvenile Fibromyalgia. *Clinical Journal of Pain, 32*, 70-81. DOI: http://dx.doi.org/10.1097/AJP.0000000000000221.

Kirk, S. & Milnes, L. (2016) An exploration of how young people and parents use online support in the context of living with cystic fibrosis. *Health Expectations, 19*, 309-321. DOI: http://dx.doi.org/10.1111/hex.12352.

Lewis, P., Klineberg, E., Towns, S., Moore, K. & Steinbeck, K. (2016) The Effects of Introducing Peer Support to Young People with a Chronic Illness. *Journal of Child and Family Studies, 25*, 2541-2553. DOI: 10.1007/s10826-016-0427-4.

MacDonald, K. & Greggans, A. (2010) ‘Cool friends’: an evaluation of a community befriending programme for young people with cystic fibrosis. *Journal of Clinical Nursing, 19*(17‐18), 2406-2414. DOI: 10.1111/j.1365-2702.2010.03294.x.

Marsac, M. L., Hildenbrand, A. K., Clawson, K., Jackson, L., Kohser, K., Barakat, L., . . . Alderfer, M. A. (2012) Acceptability and feasibility of family use of The Cellie Cancer Coping Kit. *Supportive Care in Cancer, 20*, 3315-3324. DOI: http://dx.doi.org/10.1007/s00520-012-1475-y.

Masuda, J. R., Anderson, S., Letourneau, N., Sloan Morgan, V. & Stewart, M. (2013) Reconciling Preferences and Constraints in Online Peer Support for Youth With Asthma and Allergies. *Health Promotion Practice, 14*, 741-750. DOI: 10.1177/1524839912465083.

Moola, F. J., Faulkner, G., White, L. & Kirsh, J. (2015) Kids with special hearts: the experience of children with congenital heart disease at Camp Willowood. *Qualitiative Research in Sport, Exercise and Health, 7*, 271-293. DOI: 10.1080/2159676X.2014.926968.

Muskat, B., Salter, R., Shindler, S., Porter, M. & Bitnun, A. (2016) “Here you feel like it's not taboo”: An evaluation of a pediatric hospital-based HIV support group. *Journal of HIV/AIDS & Social Services*, 1-18. DOI: http://dx.doi.org/10.1080/15381501.2015.1124310.

Nicholas, D. B., Darch, J., McNeill, T., Brister, L., O'leary, K., Berlin, D. & Roller, D. (2007) Perceptions of online support for hospitalized children and adolescents. *Social Work in Health Care, 44*, 205-223. DOI: 10.1300/J010v44n03_06.

Nicholas, D. B., Fellner, K. D., Frank, M., Small, M., Hetherington, R., Slater, R. & Daneman, D. (2012) Evaluation of an online education and support intervention for adolescents with diabetes. *Social Work in Health Care, 51*, 815-827. DOI: http://dx.doi.org/10.1080/00981389.2012.699507.

Nicholas, D. B., Williams, M. & MacLusky, I. B. (2009) Evaluating group work within a summer camp intervention for children and adolescents with asthma. *Social Work with Groups, 32*, 209-221. DOI: http://dx.doi.org/10.1080/01609510802527425.

Nieto, R., Hernandez, E., Boixados, M., Huguet, A., Beneitez, I. & McGrath, P. (2015) Testing the Feasibility of DARWeb: An Online Intervention for Children With Functional Abdominal Pain and Their Parents. *Clinical Journal of Pain, 31*, 493-503. DOI: http://dx.doi.org/10.1097/AJP.0000000000000199.

Nilsson, S., Finnstrom, B., Kokinsky, E. & Enskar, K. (2009) The use of Virtual Reality for needle-related procedural pain and distress in children and adolescents in a paediatric oncology unit. *European Journal of Oncology Nursing, 13*, 102-109. DOI: http://dx.doi.org/10.1016/j.ejon.2009.01.003.

O'Callaghan, C., Baron, A., Barry, P. & Dun, B. (2011) Music's relevance for pediatric cancer patients: a constructivist and mosaic research approach. *Supportive Care in Cancer, 19*, 779-788. DOI: http://dx.doi.org/10.1007/s00520-010-0879-9.

O'Callaghan, C., Barry, P. & Thompson, K. (2012) Music's relevance for adolescents and young adults with cancer: a constructivist research approach. *Supportive Care in Cancer, 20*, 687-697. DOI: http://dx.doi.org/10.1007/s00520-011-1104-1.

O'Callaghan, C., Dun, B., Baron, A. & Barry, P. (2013) Music's relevance for children with cancer: music therapists' qualitative clinical data-mining research. *Social Work in Health Care, 52*(2-3), 125-143. DOI: http://dx.doi.org/10.1080/00981389.2012.737904.

Reme, S. E., Archer, N. & Chalder, T. (2013) Experiences of young people who have undergone the Lightning Process to treat chronic fatigue syndrome/myalgic encephalomyelitis--a qualitative study. *British Journal of Health Psychology, 18*, 508-525. DOI: http://dx.doi.org/10.1111/j.2044-8287.2012.02093.x.

Romero, S. L. (2014) The development and impact of an online social networking community for adolescents with cystic fibrosis. *Dissertation Abstracts International: Section B: The Sciences and Engineering, 74*(10-B(E)), No Pagination Specified.

Serlachius, A., Northam, E., Frydenberg, E. & Cameron, F. (2012) Adapting a generic coping skills programme for adolescents with type 1 diabetes: a qualitative study. *Journal of Health Psychology, 17*, 313-323. DOI: http://dx.doi.org/10.1177/1359105311415559.

Shrimpton, B. J., Willis, D. J., Tongs, C. D. & Rolfo, A. G. (2013) Movie making as a cognitive distraction for paediatric patients receiving radiotherapy treatment: qualitative interview study. *BMJ Open, 3*. DOI: http://dx.doi.org/10.1136/bmjopen-2012-001666.

Sibinga, E. M., Kerrigan, D., Stewart, M., Johnson, K., Magyari, T. & Ellen, J. M. (2011) Mindfulness-based stress reduction for urban youth. *Journal of Alternative and Complementary Medicine, 17*, 213-218. DOI: http://dx.doi.org/10.1089/acm.2009.0605.

Stewart, M., Barnfather, A., Magill-Evans, J., Ray, L. & Letourneau, N. (2011b) Brief report: an online support intervention: perceptions of adolescents with physical disabilities. *Journal of Adolescence, 34*, 795-800. DOI: http://dx.doi.org/10.1016/j.adolescence.2010.04.007.

Stewart, M., Letourneau, N., Masuda, J. R., Anderson, S. & McGhan, S. (2013a) Impacts of online peer support for children with asthma and allergies: It just helps you every time you can't breathe well". *Journal of Pediatric Nursing, 28*, 439-452. DOI: http://dx.doi.org/10.1016/j.pedn.2013.01.003.

Stewart, M., Letourneau, N., Masuda, J. R., Anderson, S. & McGhan, S. (2013b) Online support for children with asthma and allergies. *Journal .of Family Nursing, 19*, 171-197. DOI: http://dx.doi.org/10.1177/1074840713483573

Stewart, M., Masuda, J. R., Letourneau, N., Anderson, S. & McGhan, S. (2011a) "I want to meet other kids like me": support needs of children with asthma and allergies. *Issues in Comprehensive Pediatric Nursing, 34*, 62-78. DOI: 10.3109/01460862.2011.572638

Stinson, J., McGrath, P., Hodnett, E., Feldman, B., Duffy, C., Huber, A., Tucker, L., Hetherington, R., Tse, S., Spiegel, L., Campillo, S., Gill, N. & White, M. (2010) Usability Testing of an Online Self-management Program for Adolescents With Juvenile Idiopathic Arthritis. *Journal of Medical Internet Research, 12*. DOI: 10.2196/jmir.1349

Stinson, J. N., Toomey, P. C., Stevens, B. J., Kagan, S., Duffy, C. M., Huber, A., Malleson, P., McGrath, P.J., Yeung, R. S. & Feldman, B. M. (2008) Asking the experts: Exploring the self‐management needs of adolescents with arthritis. *Arthritis Care and Research, 59*, 65-72. DOI: 10.1002/art.23244

Tiemens K., H. L. B. N. D. B. (2007) Evaluation of a Therapeutic Camp Program for Adolescents with a Facial Difference. *Social Work with Groups 30*, 57-71. DOI: http://dx.doi.org/10.1300/J009v30n02_06.

Weekes, D. P., Kagan, S. H., James, K. & Seboni, N. (1993) The phenomenon of hand holding as a coping strategy in adolescents experiencing treatment-related pain. *Journal of Pediatric Oncology Nursing, 10*, 19-25. DOI: 10.1177/104345429301000105.

White, L. (2014). A Therapeutic Recreation Camp for Children with Congenital Heart Disease: Examining Impact on the Psycho-social Well-being of Caregivers and their Children. [Master of Science]. University of Toronto.

White, L. C., Moola, F. J., Kirsh, J. A. & Faulkner, G. E. (2016) A Therapeutic Recreation Camp for Children with Congenital Heart Disease: Examining Impact on the Psychosocial Well-Being of Parents. *Journal of Child and Family Studies*, 1-10.

Whittemore, R., Grey, M., Lindemann, E., Ambrosino, J. & Jaser, S. (2010) Development of an Internet coping skills training program for teenagers with type 1 diabetes. *Computers, informatics, nursing: CIN, 28*, 103. DOI: http://dx.doi.org/10.1097/NCN.0b013e3181cd8199.

Wolf Bordonaro, G. P. (2005) Art therapy with hospitalized pediatric patients. *Dissertation Abstracts International Section A: Humanities and Social Sciences, 66*(5-A), 1600.

Wright, P. M., White, K. & Gaebler-Spira, D. (2004) Exploring the Relevance of the Personal and Social Responsibility Model in Adapted Physical Activity: A Collective Case Study. *Journal of Teaching in Physical Education, 23*, 71-87. DOI: https://doi.org/10.1123/jtpe.23.1.71.
