# Supplementary material for: Experiences of interventions aiming to improve the mental health and well‐being of children and young people with a long‐term physical condition: A systematic review and meta‐ethnography
Source: Child Care Health Dev. 2019 Aug 16;45(6):832–49. doi: 10.1111/cch.12708 (PMC6851835; doi:10.1111/cch.12708)
Supplement: Supplementary file 2 — Table S2: Description of Interventions [file CCH-45-832-s002.docx]

Table S2: Description of Interventions

| **First author (Date)** | **Participants** | **Mental health target of intervention** | **Other targets of intervention** | **Intervention name^a^** | **Intervention category** | **Site of intervention** | **Delivered by** | **Structure** |
| --- | --- | --- | --- | --- | --- | --- | --- | --- |
| [Ayers (2011](#_ENREF_1)) | 14 CYP with **Cystic Fibrosis** | Anxiety, distress | NA | Inhaled nitrous oxide, Play therapy, Parent support | NA | NR | NR | NR |
| [Barlow (1999](#_ENREF_2)) | 10 CYP with **Juvenile Arthritis**, 13 Parents, 7 Health professionals | Emotional impact of JA | LTC knowledge, beliefs and behaviour | Psycho-educational interventions | Psycho-educational | NA | NA | NA |
| [Barnetz (2012](#_ENREF_3)) | 32 CYP with **Type 1 Diabetes** | Acceptance and acknowledgment of diabetes, development of coping skills | Diabetes self-management | Sponsorship for Adolescents with Diabetes | Mentoring | Public places | Mentors: young adults with diabetes (20 and 30 years) | 52 2-hour sessions held weekly |
| [Barnfather (2011](#_ENREF_4));  [Stewart (2011](#_ENREF_50)b) | 22 CYP **Cerebral Palsy** (50%), or **Spina Bifida** (50%) | Emotional support, coping with stress of condition | Social support and LTC outcomes | Computer mediated support | Computer support group (with peer mentors) | Computer mediated. | 5 peer mentors (2 CP, 3 SB). 2 Psychologists advised | 25 1-1.5 hour sessions held weekly |
| [Barry (2010](#_ENREF_5)) | 10 CYP with **Cancer** | Coping, distress | NA | Music therapy CD creation (MTCD) | Music therapy | MTCDs produced in hospital waiting space. Music played in radiation therapy treatment room. | Music therapist | 1 CD/CYP, 1 session per CD. Session Duration: 10-90 minutes (CD length 20-90m) |
| [Baruch, (2010](#_ENREF_6)) | 6 CYP with **Cancer** | Psychosocial adjustment, coping, resilience, self-esteem, mastery, self-transcendence | Social integration, confidence/ mastery, uncertainty in illness | Beads of Courage Program | Narrative/ Psychosocial support/Reward programme | Paediatric cancer centre | Clinician | Duration 2.5 months-3 years. |
| [Bignall (2015](#_ENREF_8)) | 15 African American CYP with **Asthma** | Anxiety | Asthma symptoms | Relaxation/ Breathing retraining | Relaxation (Breathing) | School-based health centre + home practise | Researcher | 2 30-minute sessions once per month |
| [Bluebond-Langer (1991](#_ENREF_9)) | 50 CYP with **Cancer** | Self-esteem, coping | Peer relationships | Camp Can-Do | Camp | YMCA camp facility | Volunteer | 1-week long camp |
| [Brodeur (2005](#_ENREF_10)) | 26 Total sample: 10 CYP with LTC , 13 Parents (9 mothers, 4 fathers), 3 Siblings. | Mental health | Social support, family cohesion | Living Well Program | Family art therapy | Children's Museum | Program coordinator, clinical/ counselling psychology doctoral student, counsellors volunteers | 14 2-hour weekly sessions. 5 month gap between first and last 7 sessions. |
| [Brothers (2014](#_ENREF_11)) | 22 young women with **HIV** | Emotional wellbeing, self-esteem, managing emotions (stress, anger, negative thinking) | Control, empowerment, improve relationships, role of alcohol and drugs, reduce secondary HIV transmission | Evolution: Young women taking charge and growing stronger…a secondary prevention empowerment intervention | Group intervention/ Group therapy | Clinical sites in three cities | Intervention deliverers | 9 2-3 hours weekly sessions |
| [Bultas (2015](#_ENREF_12)) | 50 CYP with **CHD** | Self-esteem, self-efficacy, anxiety | Peer support, attitude to LTC | Overnight summer recreational camp | Camp | NR | Camp Counsellor | 1 5-day camp |
| [Burns (2010](#_ENREF_13)) | 7 CYP with **Cancer** | Reduce symptom distress, improve coping | Understanding of LTC, family relationships | Therapeutic Music Video | Music Therapy | NR | Music therapist | 6 sessions |
| [Campbell (2010](#_ENREF_14)) | 6 African Adolescents with **HIV** | Emotional/ Psychological skills, coping | Behavioural skills; managing HIV related issues, HIV education, engagement with health professionals | Looking forward project | Group Work | Non clinical environment (e.g. church hall, restaurant) in London | Clinical psychologists, HIV clinical nurse specialist | 3 1-day sessions per year |
| [Curle (2005](#_ENREF_16)) | 11 Children with LTC 12 Parents | Coping and adaptation | Social support for children and parents | Terrific Tuesday Group (TTG) | Group therapy | Specialist unit' | Clinical psychologist, occupational therapist, mental health nurses, specialist paediatric nurses, social workers | 6 to 8 weekly sessions |
| [Dennison (2010](#_ENREF_17)) | 16 CYP with **CFS**  16 Parents | Emotional responses | Managing physical symptoms and functional impairment | Family focused CBT vs Psycho-education | CBT, psychoeducation | Hospital | NR | NR |
| [Desai (2014](#_ENREF_18)) | 13 CYP with **CHD** | Coping, self-esteem, psychosocial needs | Social support, healthcare outcomes, physical needs | Therapeutic weekend camp | Camp | South-eastern USA campsite | Trained college students and community-based volunteers. 3 registered nurses, 1 paediatric cardiologist Camping facility employees | 1, 2-day camp. |
| [Docherty (2013](#_ENREF_19)) | 16 Adolescents with **Cancer** | Illness-related distress; improved coping | Family environment, perceived social support, derived meaning, resilience, quality of life | Therapeutic music video (TMV) intervention | Music Therapy | Hospitals: 6 paediatric and 3 adult hospitals across the United States | Music Therapist | 6 1-hour sessions |
| [Fair (2012](#_ENREF_20)) | 7 Adolescents with **HIV** | Mental health, problem solving, management of emotions and stress of medication side-effects | Control, physical health, adherence | Teens Out Loud creative writing groups | Creative writing (Group) | NR | Group Leaders | 8 4-hour sessions per year. Once per month in fall and spring |
| Gan (2010) | 8 Adolescents with **ABI** 14 Family members (9 parents, 1 partner, 4 siblings) | Emotional recovery, coping, anger and stress management | Education re: brain injury and effects on family, enabling independence, managing transitions, skill building, goal setting, problem-solving | Brain Injury Family Intervention for Adolescents (BIFI-A) | Family intervention/ therapy | Family home or rehabilitation centre. | 2 social workers, 1 rehabilitation counsellor | 7 (sometimes 8-9) 1-2 hour sessions held over 3.3 months (mean duration). range 1.5–5.0 months |
| [Gaysynsky (2015](#_ENREF_21)) | 43 CYP with **HIV** | Emotional support, esteem support. coping | Engagement of services with clients | Young Adult Programme (YAP) Facebook group | Online support group | Facebook | Self, group moderated by social worker from YAP | NA |
| [Gillard (2011](#_ENREF_24)) | 24 CYP with **HIV** | Coping | Transition into adulthood. Living with HIV/AIDS managing AIDS related issues | Camp Strong and Teen Forum | Camp | NR | Counsellors, medical staff, psychosocial staff, and the camp director | 1 6-day camp per year |
| [Gillard (2013](#_ENREF_23)) | 10 CYP with **Cancer** | Emotional healing and/or self-esteem | Independence, self-esteem, "normality", social inclusion recreation | Residential camp program | Camp | Camp in Southern United States | Medical staff from cancer centre, volunteers. | 1 7-day camp per year |
| [Gillard (2016](#_ENREF_22)) | 24 CYP with LTC | Psychosocial aspects | Developmental support, building skills | The Hole in the Wall Gang Camp (THITWGC) | Camp | Medical speciality camp Connecticut | NR | 1 7-day camp per year |
| [Griffiths (2015](#_ENREF_25)) | 12 CYP with **Cancer** | Self-esteem, emotional support, coping | Social support, medical knowledge, acceptance of disease, sense of belonging | Realshare online community | Online support group | NA | First 3 months: self; 2nd 3 months self+ facilitator | 2 3-month phases. 2nd phase 3-5 times a week. |
| [Hosek (2012](#_ENREF_26)) | 17 Young women with **HIV** | Mental health, psychological concerns | Coping with HIV related issues | Secondary prevention intervention for young HIV-positive women | Secondary Prevention | NA | NA | NA |
| [Jaser (2014](#_ENREF_27)) | 20 Adolescents with **Type 1 Diabetes** | Positive affect, stress coping | Adherence, Self-management | Check It! | Positive affirmation | NR for initial interview. Home | Parent and research assistants | 1 initial interview, 16 Phone calls: 2 every week |
| [Kashikar-Zuck (2016](#_ENREF_28)) | 17 Adolescents with **JFM** | Coping skills | Increase exercise participation, reduce pain, confidence | FIT Teens | CBT + Neuromuscular exercise training | Children's hospital | Psychology post-doc and master's student | 16 60-minute sessions, twice per week |
| [Kirk (2016](#_ENREF_29)) | 97 CYP with **CF** and 182 Parents posted | Emotional support | Social support, self-care | Online Support Group | Online Support Group | Online | Self | NA |
| [Lewis (2016](#_ENREF_30)) | 4 Adolescents with LTC (new members); 4 Adolescents with LTC (established members); 19 Parents of new members; 5 Co-ordinators | Self-esteem and diminish psychological distress | NA | Chronic Illness Peer Support (ChIPS) | Peer Support | Children’s Hospital | Nurse (supported by a volunteer and other medical and allied health staff members). | 8 2-hour weekly introductory sessions, session. 4 social events a year, one annual camp |
| [MacDonald (2010](#_ENREF_31)) | 5 CYP with **Cystic Fibrosis** | Self-esteem, stress | Empowerment and independence, boredom and social isolation, self-confidence, general wellbeing | Cool Friends | Befriending programme | NR | Volunteers | Frequency of meetings ranged from once to monthly across participants. Duration of befriender relationship ranged from one month-two years across participants. |
| [Marsac (2012](#_ENREF_32)) | 15 CYP with **Cancer** 15 Parents | Augment coping, decrease distress | NA | Cellie Cancer Coping Kit | Coping kit (consider education or coping strategies) | Home and hospital | Self/parent | Used Cellie independently for 4 weeks |
| [Masuda (2013](#_ENREF_33)) | 14 CYP with **Asthma and Life Threatening Allergies** 8 parents 5 peer mentors | Coping (self-efficacy, isolation) | Peer relationships | Professionally mediated online support intervention: Ability online | Online support | Online | Self, peer mentor, health promotion professionals | 12 60-minute, weekly chat sessions |
| [Moola (2015](#_ENREF_35)) | 15 CYP with **CHD** and their bereaved siblings | Psychosocial stress | Quality of Life. fun, social, skill building, isolation, independence, confidence, mastery | Camp Willowood | Camp | Camp Willowood, 200km North of Toronto | Volunteers: child survivors of chronic illnesses, doctors and nurses provide medical care | NR |
| [Muskat (2016](#_ENREF_36)) | 16 CYP with **HIV** | Anxiety and depression | Understanding of HIV related issues, social support | Open-ended, long term support groups | Support Group | Hospital for Sick Children Family-Centered HIV clinic | Social workers | Once monthly sessions. Median sessions attended: 12, range 3–20 |
| [Nicholas (2007](#_ENREF_37)) | 9 CYP with LTC | Worry, anxiety, coping | Social support, engagement with treatment, knowledge, pain management | Starbright world | Online games, education and Peer support network | Online peer dialogue with patients in over 95 children’s hospitals | Self | All used the network a minimum of three times |
| [Nicholas (2009](#_ENREF_39)) | 22 CYP with **Asthma** | Self-efficacy, coping, stress management | Social support, self-management skills, adherence barriers | Residential Summer Camp | Asthma education delivered at Summer Camp | Ontario, Canada | Paediatric health care professionals, nurse, respiratory therapist, social worker | Air force sessions: daily during 2 week long camp |
| [Nicholas (2012](#_ENREF_38)) | 15 CYP with **Type 1 diabetes** | Coping, wellbeing, stress | Social support, self-management, managing relationships | Online education and support | Online education and support | Online | Self, website moderator | 8 weekly modules |
| [Nieto (2015](#_ENREF_40)) | 15 CYP with **FAP** and their families | Stress management, catastrophizing, coping strategies | Parenting behaviours, prevention of long term disability and pain | DAR-Web | Online psychoeducation | Online | Self | 7 30-minute weekly modules, completed by parents and children separately |
| [Nilsson (2009](#_ENREF_41)) | 21 CYP with **Cancer** | Procedural distress | Procedural pain | VR game | Game/Virtual Reality/ | NR assumed hospital. Use VR during procedure | Self | Once per procedure, 6-10 minutes |
| [O'Callaghan (2011](#_ENREF_42)) | 26 CYP with **Cancer** | Mood, coping | Play activity, engagement, | Music therapy | Music therapy | Hospital (inpatient and outpatient settings) | Music therapist/self | NA. |
| [O'Callaghan (2012](#_ENREF_43)) | 12 CYP with **Cancer** | Self-esteem and stress management | Spirituality, confidence/ mastery, hope, and self-transcendence | Music therapy | Music, music therapy | NR | Music therapist | NA |
| [O'Callaghan (2013](#_ENREF_44)) | CYP with **Cancer** | Psychosocial domains, mood, well-being | Quality of life, educational, and physical domains, engagement, play, empowerment/ control | Music, including music therapy | Music therapy | 3 hospitals in Melbourne, Australia | Music Therapists | NA |
| [Reme (2013](#_ENREF_45)) | 12 CYP with **CFS** | Decrease stress | CFS symptoms | Lightning Process | The Lightning Process | NR (various) | Lightning Process Practitioners | 3 3-5 hour sessions, once per day |
| [Romero (2014](#_ENREF_46)) | 74 CYP with **Cystic Fibrosis** | Psychological well-being | Disease management, social support |  | Online education and support | Online | Self | NA |
| [Serlachius (2012](#_ENREF_47)) | 13 Adolescents with **Type 1 Diabetes** | Coping/diabetes stress | Glycaemic control | Best of Coping (BOC) | Coping skills intervention | NR | NR | 10 Sessions |
| [Shrimpton (2013](#_ENREF_48)) | 40 CYP with **Cancer** | Fear of radiotherapy/ procedural distress, anxiety | Comfort, understand radiotherapy process and communicate this to people around them | Movie making programme | Creative therapy/ Narrative therapy | Peter MacCallum Cancer Centre, Melbourne, Australia. | Radiation therapists | NR |
| [Sibinga (2011](#_ENREF_49)) | 5 CYP with **HIV** | Stress and psychological distress, rumination, anxiety | Improved physical health as result of decreased stress (HRQOL) enhancing non-judgmental present-focused awareness | Mindfulness-based stress reduction (MBSR) | Mindfulness-based stress reduction | Clinic assumed | MBSR instructor | 9 Weekly sessions |
| [Stewart (2011](#_ENREF_53)a) | 20 CYP with **Asthma** and/or **Severe Allergies** | Coping, reduce stresses | Social support, loneliness | Psychosocial Support | Games and Online peer support | Online | Facilitators: 3 female, 2 male mentors with cerebral palsy or spina bifida, 2 psychologists. | NA |
| [Stewart (2013](#_ENREF_52)a)  [Stewart 2013](#_ENREF_51)b) | 27 CYP with **Asthma** and/or **Severe Allergies** | Coping | Education, social support | Online support intervention | Online Support Group/Mentoring | Online | 5 Peer mentors with asthma and allergies (13-20 years old), a psychologist | 8 45-120 minute weekly sessions, 1-5 hours per week |
| [Stinson (2008](#_ENREF_55)) | 36 CYP with **Juvenile Idiopathic Arthritis** | Self-efficacy, Psychological consequences | Knowledge, self-management behaviours | Web-based program of self-management | Online support | NR | NR | NR |
| [Stinson (2010](#_ENREF_54)) | 19 CYP with **Juvenile Idiopathic Arthritis** 19 Parents | Stress | Social support, symptom management, JIA-specific education, | Teens Taking Charge: Managing Arthritis Online | Online self-management /education programme | Online | Self | 1 40-minute trial session |
| [Tiemens (2007](#_ENREF_57)) | 4 CYP with **craniofacial difference** | Self-esteem | Social support, reducing isolation, task mastery, self-reflection, therapeutic gain | AboutFace | Camp | Outdoor recreational facility | Staff at recreational facility, social workers | 1 weekend |
| [Weekes (1993](#_ENREF_58)) | 10 CYP with **Cancer** 10 CYP with **Renal Failure** | Coping | NA | Hand-holding | Hand-holding | Outpatient hospital setting | Parent/ Clinician (nurse) | NA |
| [White (2014](#_ENREF_59)) [White (2016](#_ENREF_60)) | 9 CYP with **CHD** | Stress reduction | Social relationships, belonging and acceptance | Camp Oki | Camp | Camp Oki Canada | Staff and volunteers at Hospital for Sick Children | 1 7-day camp, once a year |
| [Whittemore (2010](#_ENREF_61)) | 13 Adolescents with **Type 1 Diabetes** | Coping skills, stress management, self-efficacy | Diabetes management, self-talk, social relationships | TEENCOPE and the Managing Diabetes Internet interventions programme | Internet coping skills training program | Online, otherwise not reported. | NR | TEENCOPE: 5 sessions, Managing Diabetes: 4 weekly sessions. |
| [Wolf Bordonaro (2005](#_ENREF_62)) | 3 CYP with **Sickle Cell Disease** | Anxiety, stress management | Control, independence, self-responsibility | Art Therapy | Art Therapy | Patients hospital room or hospital activity room. | Art Therapist | 3 1-hour art therapy sessions within 1 or 2 days |
| [Wright (2004](#_ENREF_63)) | 12 CYP with **Cerebral Palsy** | Self-esteem, self-efficacy | Wellbeing, self-determination, empowerment, personal and social responsibility, physical/social skills, empowerment, self-worth | Personal and Social Responsibility Model (PSRM)/Developmental Martial Arts Program (DMAP) | Physical activity | Assumed paediatric outpatient unit | Martial arts instructor/researcher assistant instructor physical therapists | 13 45-minute weekly sessions |

^a^Intervention or components named in study

Key: BIFI-A = Brain Injury Family Intervention for Adolescents, CBT = Cognitive Behavioural Therapy, CFS = Chronic Fatigue Syndrome, CHD = Congenital Heart Disease, ChIPS = Chronic Illness Peer Support, DMAP = Developmental Martial Arts Program, FAP = Functional Abdominal Pain, HRQOL = Health Related Quality of Life, JFM = Juvenile Fibromyalgia, MBSR = Mindfulness Based Stress Reduction, MTCD = Music Therapy CD Creation, NA = Not Applicable, NR = Not Reported, PSRM = Personal and Social Responsibility Model, TMV = Therapeutic Music Video, TTG = Terrific Tuesday Group, VR = Virtual Reality , YAP = Young Adults Programme , YMCA = Young Men’s Christian Association.
