# Supplementary material for: Experiences of interventions aiming to improve the mental health and well‐being of children and young people with a long‐term physical condition: A systematic review and meta‐ethnography
Source: Child Care Health Dev. 2019 Aug 16;45(6):832–49. doi: 10.1111/cch.12708 (PMC6851835; doi:10.1111/cch.12708)
Supplement: Supplementary file 3 — Table S3: Quality Appraisal of Included Studies [file CCH-45-832-s003.docx]

Table S3: Quality Appraisal of Included Studies

|  | Is the research question clear? | Is the theoretical or ideological perspective of the author (or funder) explicit? | Has this influenced the study design, methods or  research findings? | Is the study design appropriate to answer the question? | Is the context or setting adequately described? | Is the sample adequate to explore the range of subjects  and settings, and has it been drawn from an  appropriate population? | Was the data collection adequately described? | Was data collection rigorously conducted to  ensure confidence in the findings? | Was there evidence that the data analysis was rigorously conducted to  ensure confidence in the findings? | Are the findings substantiated by the data? | Has consideration been given to any limitations of  the methods or data that may have affected the results? | Do any claims to generalisability follow logically and theoretically from  the data? | Have ethical issues been addressed and confidentiality respected? | Are the interventions of interest clearly described? |
| --- | --- | --- | --- | --- | --- | --- | --- | --- | --- | --- | --- | --- | --- | --- |
| Ayers (2011) | Y | N | CT^a^ | Y | Y | Y | Y | Y | Y | Y | Y | Y | Y | N |
| Barlow (1999) | Y | N | CT | Y | Y | Y | Y | Y | Y | Y | Y | Y | N | N |
| Barnetz (2012) | Y | N | CT | Y | N | Y | Y | N | Y | Y | Y | Y | CT | Y |
| Barnfather (2011) | Y | Y | Y | Y | Y | Y | Y | CT | Y | Y | N | Y | Y | Y |
| Barry (2010) | Y | N | CT | Y | Y | Y | Y | Y | Y | Y | Y | Y | Y | Y |
| Baruch (2010) | Y | Y | Y | Y | Y | Y | Y | Y | Y | Y | Y | N | Y | N |
| Bignall (2015) | Y | N | CT | CT | N | Y | N | CT | N | Y | Y | N | N | Y |
| Bluebond-Langer (1991) | N | N | CT | Y | Y | Y | Y | CT | N | Y | N | Y | Y | Y |
| Brodeur (2005) | Y | Y | Y | Y | Y | Y | Y | Y | Y | Y | Y | Y | Y | Y |
| Brothers (2014) | Y | N | CT | Y | N | Y | N | Y | N | Y | Y | N | N | Y |
| Bultas (2015) | Y | N | CT | Y | N | Y | Y | CT | Y | Y | Y | N | Y | N |
| Burns (2010) | Y | Y | Y | Y | Y | Y | Y | Y | Y | Y | N | Y | Y | Y |
| Campbell (2010) | Y | N | CT | Y | Y | N | Y | Y | Y | Y | Y | Y | N | N |
| Curle (2005) | Y | Y | Y | Y | Y | Y | Y | N | Y | Y | Y | Y | Y | Y |
| Dennison (2010) | Y | N | CT | Y | Y | Y | Y | Y | Y | Y | Y | Y | Y | N |
| Desai (2014) | Y | N | CT | Y | Y | Y | Y | Y | Y | Y | Y | Y | Y | Y |
| Docherty (2013) | Y | N | CT | Y | N | Y | Y | Y | Y | Y | Y | N | Y | N |
| Fair (2012) | N | N | CT | Y | Y | Y | Y | CT | Y | Y | Y | Y | N | Y |
| Gan (2010) | Y | N | CT | Y | Y | Y | Y | CT | N | Y | Y | Y | Y | Y |
| Gaysynsky (2015) | Y | N | CT | Y | Y | Y | Y | Y | Y | Y | Y | Y | N | Y |
| Gillard (2011) | Y | N | CT | Y | N | Y | Y | Y | Y | Y | N | Y | Y | Y |
| Gillard (2013) | Y | N | CT | Y | N | Y | Y | Y | Y | Y | Y | Y | N | Y |
| Gillard (2016) | Y | Y | Y | Y | N | Y | Y | N | Y | Y | Y | N | Y | N |
| Griffiths (2015) | Y | Y | Y | Y | N | Y | Y | CT | Y | Y | Y | Y | Y | N |
| Hosek (2012) | Y | Y | Y | Y | N | Y | Y | Y | Y | Y | Y | N | N | N |
| Jaser (2014) | Y | N | CT | Y | N | Y | Y | Y | Y | Y | Y | Y | N | Y |
| Kashikar-Zuck (2016) | Y | N | CT | Y | Y | Y | Y | Y | Y | Y | N | Y | Y | Y |
| Kirk (2016) | Y | N | CT | Y | N | Y | Y | Y | N | Y | N | Y | Y | N |
| Lewis (2016) | Y | N | CT | Y | Y | Y | Y | Y | Y | Y | N | Y | Y | Y |
| MacDonald (2010) | Y | N | CT | Y | N | Y | N | Y | Y | Y | Y | N | Y | N |
| Marsac (2012) | Y | N | CT | Y | N | Y | Y | Y | Y | Y | Y | Y | N | Y |
| Masuda (2013) | Y | N | CT | Y | N | Y | Y | CT | N | Y | N | N | Y | Y |
| Moola (2015) | Y | Y | Y | Y | Y | Y | Y | Y | Y | Y | N | Y | Y | Y |
| Muskat (2016) | Y | N | CT | Y | N | Y | Y | Y | Y | Y | Y | Y | Y | N |
| Nicholas (2007) | Y | N | CT | N | N | Y | N | Y | Y | Y | N | N | CT | Y |
| Nicholas (2009) | N | N | CT | Y | N | Y | N | CT | Y | N | Y | CT | Y | Y |
| Nicholas (2012) | N | N | CT | Y | N | Y | Y | Y | Y | Y | Y | Y | Y | Y |
| Nieto (2015) | Y | N | CT | Y | N | Y | Y | Y | Y | Y | Y | Y | Y | Y |
| Nilsson 2009) | Y | N | CT | Y | Y | Y | Y | CT | Y | Y | N | Y | Y | Y |
| O'Callaghan (2011) | Y | Y | Y | Y | Y | Y | Y | Y | Y | Y | Y | Y | Y | N |
| O'Callaghan (2012) | N | N | CT | Y | Y | Y | Y | Y | Y | Y | N | Y | Y | N |
| O'Callaghan (2013) | Y | N | CT | Y | Y | N | Y | Y | Y | Y | Y | N | Y | N |
| Reme (2013) | Y | N | CT | Y | Y | Y | Y | Y | Y | Y | Y | Y | Y | Y |
| Romero (2013) | Y | N | CT | Y | Y | Y | Y | Y | Y | Y | Y | Y | Y | N |
| Serlachius 2012) | N | Y | Y | Y | N | Y | Y | Y | Y | Y | Y | Y | Y | Y |
| Shrimpton (2013) | Y | N | CT | Y | N | N | Y | Y | Y | Y | Y | Y | Y | N |
| Sibinga (2011) | Y | N | CT | Y | N | N | Y | Y | N | N | Y | Y | Y | Y |
| Stewart (2013a) | Y | Y | Y | Y | Y | Y | Y | Y | Y | Y | N | Y | Y | Y |
| Stewart (2013b) | Y | N | CT | Y | Y | Y | Y | Y | Y | Y | Y | Y | Y | Y |
| Stewart (2011a) | Y | N | CT | Y | N | Y | Y | Y | Y | Y | N | N | Y | N |
| Stewart (2011b) | Y | N | CT | Y | N | Y | N | CT | N | Y | N | Y | CT | N |
| Stinson (2010) | Y | N | CT | Y | Y | Y | Y | Y | N | Y | Y | Y | Y | Y |
| Stinson (2008) | Y | N | CT | Y | Y | Y | Y | Y | Y | Y | Y | Y | Y | N |
| Tiemens (2007) | Y | N | CT | Y | N | Y | N | Y | Y | Y | Y | Y | N | Y |
| Weekes (1993) | Y | Y | Y | Y | Y | Y | Y | Y | Y | Y | N | N | N | N |
| White (2016) | Y | N | CT | N | Y | Y | Y | Y | Y | Y | Y | Y | Y | Y |
| White (2014) | N | Y | Y | N | Y | N | Y | Y | Y | Y | Y | Y | Y | Y |
| Whittemore (2010) | Y | N | CT | Y | N | N | N | N | N | Y | N | N | N | Y |
| Wolf-Bordonaro (2003) | Y | Y | Y | N | N | N | Y | CT | N | N | Y | N | Y | Y |
| Wright (2004) | Y | N | CT | Y | N | N | Y | Y | Y | Y | Y | N | Y | Y |

Y = yes; N = no; CT = cannot tell; green cell denotes positive score; yellow cell denotes unclear or moderate score; red cell denotes negative score.
