# Supplementary material for: Experiences of interventions aiming to improve the mental health and well‐being of children and young people with a long‐term physical condition: A systematic review and meta‐ethnography
Source: Child Care Health Dev. 2019 Aug 16;45(6):832–49. doi: 10.1111/cch.12708 (PMC6851835; doi:10.1111/cch.12708)
Supplement: Supplementary file 4 — Table S4: Articles which contribute towards each overarching construct and subtheme [file CCH-45-832-s004.docx]

Table S4: Articles which contribute towards each overarching construct and subtheme.

| Construct | Theme | Number of articles contributing to theme | Articles |  |
| --- | --- | --- | --- | --- |
| Therapeutic Foundation | Safe space | 19 | ^Ayers et al., 2011; Barnetz & Feigin, 2012; Dennison et al., 2010; Desai et al., 2014; Fair et al., 2012; A. Gillard et al., 2011; Griffiths et al., 2015; Lewis et al., 2016; Moola et al., 2015; Muskat et al., 2016; Nicholas et al., 2007; O'Callaghan et al., 2013; Serlachius et al., 2012; Stewart et al., 2013b; Tiemens et al., 2007; Weekes et al., 1993; L. White, 2014; Whittemore et al., 2010; Wolf Bordonaro, 2005^ |  |
|  | Unconstrained | 28 | ^Barnetz & Feigin, 2012; Barnfather et al., 2011; Bluebond-Langer et al., 1991; Brodeur, 2005; Bultas et al., 2015; Campbell et al., 2010; Dennison et al., 2010; Desai et al., 2014; Docherty et al., 2013; Fair et al., 2012; A. Gillard & Allsop, 2016; Ann Gillard & Watts, 2013; A. Gillard et al., 2011; Kirk & Milnes, 2016; Lewis et al., 2016; MacDonald & Greggans, 2010; Moola et al., 2015; Muskat et al., 2016; Nicholas et al., 2007; Nicholas et al., 2009; O'Callaghan et al., 2011; O'Callaghan et al., 2012; O'Callaghan et al., 2013; Stewart et al., 2013b; Tiemens et al., 2007; L. White, 2014; L. C. White et al., 2016; Wright et al., 2004^ |  |
|  | Therapeutic relationships | 42 | ^Ayers et al., 2011; Barnetz & Feigin, 2012; Barnfather et al., 2011; Baruch, 2010; Bignall et al., 2015; Bluebond-Langer et al., 1991; Brodeur, 2005; Brothers et al., 2014; Dennison et al., 2010; Desai et al., 2014; Docherty et al., 2013; Fair et al., 2012; Gan et al., 2010; Gaysynsky et al., 2015; A. Gillard & Allsop, 2016; Ann Gillard & Watts, 2013; A. Gillard et al., 2011; Griffiths et al., 2015; Hosek et al., 2012; Kashikar-Zuck et al., 2016; Kirk & Milnes, 2016; Lewis et al., 2016; MacDonald & Greggans, 2010; Moola et al., 2015; Muskat et al., 2016; Nicholas et al., 2007; Nicholas et al., 2012; Nicholas et al., 2009; O'Callaghan et al., 2011; O'Callaghan et al., 2012; O'Callaghan et al., 2013; Reme et al., 2013; Romero, 2014; Serlachius et al., 2012; Shrimpton et al., 2013; Stewart, Barnfather, et al., 2011; Stewart et al., 2013b; Stewart, Masuda, et al., 2011; Tiemens et al., 2007; L. White, 2014; Whittemore et al., 2010; Wolf Bordonaro, 2005^ |  |
| Social Support | I am not alone | 46 | ^Barlow et al., 1999; Barnetz & Feigin, 2012; Barnfather et al., 2011; Baruch, 2010; Bluebond-Langer et al., 1991; Brodeur, 2005; Brothers et al., 2014; Bultas et al., 2015; Campbell et al., 2010; Curle et al., 2005; Dennison et al., 2010; Desai et al., 2014; Docherty et al., 2013; Fair et al., 2012; Gan et al., 2010; Gaysynsky et al., 2015; A. Gillard & Allsop, 2016; Ann Gillard & Watts, 2013; A. Gillard et al., 2011; Griffiths et al., 2015; Hosek et al., 2012; Kashikar-Zuck et al., 2016; Kirk & Milnes, 2016; Lewis et al., 2016; MacDonald & Greggans, 2010; Marsac et al., 2012; Masuda et al., 2013; Moola et al., 2015; Muskat et al., 2016; Nicholas et al., 2007; Nicholas et al., 2012; Nicholas et al., 2009; Nieto et al., 2015; O'Callaghan et al., 2012; Reme et al., 2013; Serlachius et al., 2012; Stewart, Barnfather, et al., 2011; Stewart et al., 2013a, 2013b; Stewart, Masuda, et al., 2011; J. N. Stinson et al., 2008; Tiemens et al., 2007; L. White, 2014; L. C. White et al., 2016; Whittemore et al., 2010; Wright et al., 2004^ |  |
|  | Improved relationships | 34 | ^(Barlow et al., 1999; Barnetz & Feigin, 2012; Barry et al., 2010; Baruch, 2010; Bignall et al., 2015; Bluebond-Langer et al., 1991; Brodeur, 2005; Burns et al., 2010; Curle et al., 2005; Desai et al., 2014; Docherty et al., 2013; Fair et al., 2012; Gan et al., 2010; Gaysynsky et al., 2015; Ann Gillard & Watts, 2013; A. Gillard et al., 2011; Hosek et al., 2012; Jaser et al., 2014; Lewis et al., 2016; MacDonald & Greggans, 2010; Marsac et al., 2012; Nicholas et al., 2007; O'Callaghan et al., 2011; O'Callaghan et al., 2012; O'Callaghan et al., 2013; Serlachius et al., 2012; Shrimpton et al., 2013; Sibinga et al., 2011; Stewart, Barnfather, et al., 2011; Stewart et al., 2013a, 2013b; J. N. Stinson et al., 2008; Wolf Bordonaro, 2005; Wright et al., 2004^ |  |
| A Hopeful Alternative | Hope and inspiration | 30 | ^Barlow et al., 1999; Barnetz & Feigin, 2012; Barnfather et al., 2011; Baruch, 2010; Bluebond-Langer et al., 1991; Brodeur, 2005; Bultas et al., 2015; Burns et al., 2010; Campbell et al., 2010; Dennison et al., 2010; Fair et al., 2012; A. Gillard & Allsop, 2016; Ann Gillard & Watts, 2013; A. Gillard et al., 2011; Hosek et al., 2012; Kashikar-Zuck et al., 2016; Kirk & Milnes, 2016; Lewis et al., 2016; Moola et al., 2015; Nicholas et al., 2009; O'Callaghan et al., 2012; Stewart, Barnfather, et al., 2011; Stewart et al., 2013a, 2013b; J. Stinson et al., 2010; J. N. Stinson et al., 2008; Weekes et al., 1993; L. White, 2014; Wolf Bordonaro, 2005; Wright et al., 2004^ |  |
|  | More than just my illness | 33 | ^Barlow et al., 1999; Barnetz & Feigin, 2012; Barnfather et al., 2011; Brothers et al., 2014; Burns et al., 2010; Campbell et al., 2010; Dennison et al., 2010; Desai et al., 2014; Fair et al., 2012; Gan et al., 2010; A. Gillard & Allsop, 2016; Ann Gillard & Watts, 2013; A. Gillard et al., 2011; Griffiths et al., 2015; Hosek et al., 2012; Kashikar-Zuck et al., 2016; Kirk & Milnes, 2016; Moola et al., 2015; Muskat et al., 2016; Nicholas et al., 2007; Nicholas et al., 2012; O'Callaghan et al., 2012; O'Callaghan et al., 2013; Reme et al., 2013; Romero, 2014; Serlachius et al., 2012; Shrimpton et al., 2013; Stewart, Barnfather, et al., 2011; Stewart et al.; J. N. Stinson et al., 2008; L. White, 2014; Whittemore et al., 2010; Wolf Bordonaro, 2005^ |  |
|  | Being normal with an LTC | 39 | ^Barlow et al., 1999; Barnetz & Feigin, 2012; Baruch, 2010; Bluebond-Langer et al., 1991; Brodeur, 2005; Bultas et al., 2015; Burns et al., 2010; Campbell et al., 2010; Dennison et al., 2010; Desai et al., 2014; Docherty et al., 2013; Fair et al., 2012; A. Gillard & Allsop, 2016; Ann Gillard & Watts, 2013; A. Gillard et al., 2011; Griffiths et al., 2015; Hosek et al., 2012; Kashikar-Zuck et al., 2016; Kirk & Milnes, 2016; Lewis et al., 2016; Marsac et al., 2012; Moola et al., 2015; Muskat et al., 2016; Nicholas et al., 2007; Nicholas et al., 2012; Nicholas et al.; O'Callaghan et al., 2011; O'Callaghan et al., 2012; O'Callaghan et al., 2013; Reme et al., 2013; Shrimpton et al., 2013; Sibinga et al., 2011; Stewart, Barnfather, et al., 2011; Stewart et al., 2013a, 2013b; J. N. Stinson et al., 2008; L. White, 2014; Wolf Bordonaro, 2005; Wright et al., 2004^ |  |
|  | I have something to offer | 33 | ^Barlow et al., 1999; Barnetz & Feigin, 2012; Barnfather et al., 2011; Bignall et al., 2015; Bluebond-Langer et al., 1991; Brodeur, 2005; Burns et al., 2010; Desai et al., 2014; Ann Gillard & Watts, 2013; A. Gillard et al., 2011; Griffiths et al., 2015; Hosek et al., 2012; Kashikar-Zuck et al., 2016; Kirk & Milnes, 2016; Lewis et al., 2016; MacDonald & Greggans, 2010; Marsac et al., 2012; Masuda et al., 2013; Muskat et al., 2016; Nicholas et al., 2007; Nieto et al., 2015; O'Callaghan et al., 2011; Serlachius et al., 2012; Shrimpton et al., 2013; Stewart, Barnfather, et al., 2011; Stewart et al., 2013b; Stinson et al., 2010; J. N. Stinson et al., 2008; Tiemens et al., 2007; Whittemore et al., 2010; Wright et al., 2004^ |  |
| Empowerment | Self-management | 49 | ^Barlow et al., 1999; Barnetz & Feigin, 2012; Barry et al., 2010; Baruch, 2010; Bignall et al., 2015; Bluebond-Langer et al., 1991; Brodeur, 2005; Brothers et al., 2014; Burns et al., 2010; Campbell et al., 2010; Curle et al., 2005; Dennison et al., 2010; Desai et al., 2014; Docherty et al., 2013; Fair et al., 2012; Gan et al., 2010; Ann Gillard & Watts, 2013; A. Gillard et al., 2011; Hosek et al., 2012; Jaser et al., 2014; Kashikar-Zuck et al., 2016; Kirk & Milnes, 2016; Lewis et al., 2016; MacDonald & Greggans, 2010; Marsac et al., 2012; Moola et al., 2015; Muskat et al., 2016; Nicholas et al., 2007; Nicholas et al., 2012; Nicholas et al., 2009; Nieto et al., 2015; Nilsson et al., 2009; O'Callaghan et al., 2011; O'Callaghan et al., 2012; O'Callaghan et al., 2013; Reme et al., 2013; Serlachius et al., 2012; Shrimpton et al., 2013; Sibinga et al., 2011; Stewart, Barnfather, et al., 2011; Stewart et al., 2013a, 2013b; Stewart, Masuda, et al., 2011; Stinson et al., 2010; J. N. Stinson et al., 2008; Weekes et al., 1993; L. White, 2014; Whittemore et al., 2010; Wolf Bordonaro, 2005^ |  |
|  | Empowerment | 47 | ^Ayers et al., 2011; Barlow et al., 1999; Barry et al., 2010; Bignall et al., 2015; Bluebond-Langer et al., 1991; Brodeur, 2005; Brothers et al., 2014; Bultas et al., 2015; Burns et al., 2010; Campbell et al., 2010; Dennison et al., 2010; Desai et al., 2014; Docherty et al., 2013; Fair et al., 2012; Gan et al., 2010; A. Gillard & Allsop, 2016; Ann Gillard & Watts, 2013; A. Gillard et al., 2011; Hosek et al., 2012; Jaser et al., 2014; Kashikar-Zuck et al., 2016; Kirk & Milnes, 2016; Lewis et al., 2016; MacDonald & Greggans, 2010; Masuda et al., 2013; Moola et al., 2015; Muskat et al., 2016; Nicholas et al., 2007; Nicholas et al., 2009; O'Callaghan et al., 2011; O'Callaghan et al., 2012; O'Callaghan et al., 2013; Reme et al., 2013; Serlachius et al., 2012; Shrimpton et al., 2013; Sibinga et al., 2011; Stewart, Barnfather, et al., 2011; Stewart et al., 2013a, 2013b; J. Stinson et al., 2010; J. N. Stinson et al., 2008; Tiemens et al., 2007; Weekes et al., 1993; L. White, 2014; L. C. White et al., 2016; Wolf Bordonaro, 2005; Wright et al., 2004^ |  |
|  | Self-Esteem | 27 | ^Barnetz & Feigin, 2012; Barry et al., 2010; Baruch, 2010; Brodeur, 2005; Brothers et al., 2014; Burns et al., 2010; Campbell et al., 2010; Dennison et al., 2010; Desai et al., 2014; Gan et al., 2010; Gaysynsky et al., 2015; A. Gillard & Allsop, 2016; Ann Gillard & Watts, 2013; A. Gillard et al., 2011; Hosek et al., 2012; Jaser et al., 2014; Kashikar-Zuck et al., 2016; Kirk & Milnes, 2016; Nicholas et al., 2007; Shrimpton et al., 2013; Stewart, Barnfather, et al., 2011; Stewart et al., 2013a, 2013b; Tiemens et al., 2007; L. White, 2014; Whittemore et al., 2010; Wright et al., 2004^ |  |
| Getting In and Staying In | Availability | 30 | ^Barlow et al., 1999; Barnetz & Feigin, 2012; Barnfather et al., 2011; Campbell et al., 2010; Dennison et al., 2010; Desai et al., 2014; Docherty et al., 2013; Fair et al., 2012; Gan et al., 2010; Ann Gillard & Watts, 2013; A. Gillard et al., 2011; Griffiths et al., 2015; Hosek et al., 2012; Kashikar-Zuck et al., 2016; Lewis et al., 2016; MacDonald & Greggans, 2010; Masuda et al., 2013; Muskat et al., 2016; Nicholas et al., 2007; Nicholas et al., 2009; O'Callaghan et al., 2011; O'Callaghan et al., 2012; Reme et al., 2013; Stewart et al., 2013b; J. N. Stinson et al., 2008; Weekes et al., 1993; L. White, 2014; L. C. White et al., 2016; Whittemore et al., 2010; Wolf Bordonaro, 2005^ |  |
|  | Accessibility | 52 | ^Barlow et al., 1999; Barnetz & Feigin, 2012; Barnfather et al., 2011; Barry et al., 2010; Baruch, 2010; Bignall et al., 2015; Brodeur, 2005; Brothers et al., 2014; Bultas et al., 2015; Burns et al., 2010; Campbell et al., 2010; Dennison et al., 2010; Desai et al., 2014; Docherty et al., 2013; Fair et al., 2012; Gan et al., 2010; Gaysynsky et al., 2015; A. Gillard & Allsop, 2016; Ann Gillard & Watts, 2013; A. Gillard et al., 2011;Griffiths et al., 2015; Hosek et al., 2012; Jaser et al., 2014; Kashikar-Zuck et al., 2016; Kirk & Milnes, 2016; Lewis et al., 2016; MacDonald & Greggans, 2010; Marsac et al., 2012; Masuda et al., 2013; Moola et al., 2015; Muskat et al., 2016; Nicholas et al., 2007; Nicholas et al., 2012; Nicholas et al., 2009; O'Callaghan et al., 2011; O'Callaghan et al., 2012; O'Callaghan et al., 2013; Reme et al., 2013; Romero, 2014; Serlachius et al., 2012; Shrimpton et al., 2013; Stewart, Barnfather, et al., 2011; Stewart et al., 2013b; Stewart, Masuda, et al., 2011; J. Stinson et al.; J. N. Stinson et al., 2008; Tiemens et al., 2007; L. White, 2014; L. C. White et al., 2016; Whittemore et al., 2010; Wolf Bordonaro, 2005; Wright et al., 2004^ |  |
|  | Engagement | 54 | ^Barlow et al., 1999; Barnetz & Feigin, 2012; Barnfather et al., 2011; Barry et al., 2010; Baruch, 2010; Bignall et al., 2015; Bluebond-Langer et al., 1991; Brodeur, 2005; Brothers et al., 2014; Bultas et al., 2015; Burns et al., 2010; Campbell et al., 2010; Curle et al., 2005; Dennison et al., 2010; Desai et al., 2014; Fair et al., 2012; Gan et al., 2010; Gaysynsky et al., 2015; A. Gillard & Allsop, 2016; Ann Gillard & Watts, 2013; A. Gillard et al., 2011; Griffiths et al., 2015; Hosek et al., 2012; Jaser et al., 2014; Kashikar-Zuck et al., 2016; Kirk & Milnes, 2016; Lewis et al., 2016; MacDonald & Greggans, 2010; Marsac et al., 2012; Masuda et al., 2013; Moola et al., 2015; Nicholas et al., 2007; Nicholas et al., 2012; Nicholas et al., 2009; Nilsson et al., 2009; O'Callaghan et al., 2011; O'Callaghan et al., 2012; O'Callaghan et al., 2013; Reme et al., 2013; Romero, 2014; Shrimpton et al., 2013; Sibinga et al., 2011; Stewart, Barnfather, et al., 2011; Stewart et al., 2013a, 2013b; Stewart, Masuda, et al., 2011; J. Stinson et al., 2010; J. N. Stinson et al., 2008; Tiemens et al., 2007; Weekes et al., 1993; L. White, 2014; Whittemore et al., 2010; Wolf Bordonaro, 2005; Wright et al., 2004^ |  |
|  | Sustaining | 42 | ^Ayers et al., 2011; Barlow et al., 1999; Barnetz & Feigin, 2012; Barry et al., 2010; Baruch, 2010; Bignall et al., 2015; Bluebond-Langer et al., 1991; Brodeur, 2005; Brothers et al., 2014; Bultas et al., 2015; Desai et al., 2014; Fair et al., 2012; Gan et al., 2010; A. Gillard & Allsop, 2016; Ann Gillard & Watts, 2013; A. Gillard et al., 2011; Griffiths et al., 2015; Jaser et al., 2014; Kashikar-Zuck et al., 2016; Kirk & Milnes, 2016; Lewis et al., 2016; MacDonald & Greggans, 2010; Marsac et al., 2012; Moola et al., 2015; Nicholas et al., 2007; Nicholas et al., 2009; Nieto et al., 2015; O'Callaghan et al., 2011; O'Callaghan et al., 2012; O'Callaghan et al., 2013; Reme et al., 2013; Shrimpton et al., 2013; Sibinga et al., 2011; Stewart, Barnfather, et al., 2011; Stewart et al., 2013a, 2013b; J. N. Stinson et al., 2008; Tiemens et al., 2007; L. White, 2014; L. C. White et al., 2016; Whittemore et al., 2010; Wolf Bordonaro, 2005^ | |
